# Parsing genetically influenced risk pathways: genetic loci impact problematic alcohol use via externalizing and specific risk

**DOI:** 10.1038/s41398-022-02171-x

**Published:** 2022-09-30

**Authors:** Peter B. Barr, Travis T. Mallard, Sandra Sanchez-Roige, Holly E. Poore, Richard Karlsson Linnér, COGA Collaborators, Bernice Porjesz, Victor Hesselbrock, Tatiana Foroud, Arpana Agrawal, Danielle Dick, Howard J. Edenberg, John Nurrnberger, Yunlong Liu, Samuel Kuperman, John Kramer, Jacquelyn Meyers, Chella Kamarajan, Ashwini Pandey, Laura Bierut, John Rice, Kathleen Bucholz, Marc Schuckit, Jay Tischfield, Ronald Hart, Jessica Salvatore, Laura Almasy, Alison Goate, Manav Kapoor, Paul Slesinger, Denise Scott, Lance Bauer, Leah Wetherill, Xiaolong Xuei, Dongbing Lai, Sean O’Connor, Martin Plawecki, Laura Acion, Grace Chan, David B. Chorlian, Jian Zhang, Sivan Kinreich, Gayathri Pandey, Michael Chao, Andrey Anokhin, Vivia McCutcheon, Scott Saccone, Fazil Aliev, Hemin Chin, Abbas Parsian, Irwin D. Waldman, Abraham A. Palmer, K. Paige Harden, Danielle M. Dick

**Affiliations:** 1 Department of Psychiatry & Behavioral Sciences, SUNY Downstate Health Sciences University, Brooklyn, NY USA; 2 VA New York Harbor Healthcare System, Brooklyn, NY, USA; 3 Psychiatric and Neurodevelopmental Genetics Unit, Center for Genomic Medicine, Massachusetts General Hospital, Boston, MA USA; 4 Department of Psychiatry, Harvard Medical School, Boston, MA USA; 5 Department of Psychiatry, University of California San Diego, La Jolla, CA USA; 6 Division of Genetic Medicine, Vanderbilt University Medical Center, Nashville, TN USA; 7 Department of Psychiatry, Robert Wood Johnson Medical School, Rutgers University, Piscataway, NJ USA; 8 Rutgers Addiction Research Center, Rutgers University, Piscataway, NJ USA; 9 Department of Economics, Leiden University, Leiden, The Netherlands; 10 Department of Psychology, Emory University, Atlanta, GA USA; 11 Center for Computational and Quantitative Genetics, Emory University, Atlanta, GA USA; 12 Institute for Genomic Medicine, University of California San Diego, La Jolla, CA USA; 13 Department of Psychology, University of Texas at Austin, Austin, TX USA; 14 Population Research Center, University of Texas at Austin, Austin, TX USA; 15 Henri Begleiter Neurodynamics Lab, Department of Psychiatry, State University of New York, Downstate Medical Center, Brooklyn, NY USA; 16 Department of Psychiatry, University of Connecticut School of Medicine, Farmington, CT USA; 17 Department of Medical and Molecular Genetics, Indiana University School of Medicine, Indianapolis, IN USA; 18 Department of Psychiatry, Washington University School of Medicine, St. Louis, MO USA; 19 Department of Psychiatry, Rutgers Robert Wood Johnson Medical School, Piscataway, NJ USA; 20 Rutgers Addiction Research Center, Brain Health Institute, Rutgers Biomedical and Health Sciences, Newark, USA; 21 Department of Biochemistry and Molecular Biology, Indiana University School of Medicine, Indianapolis, IN USA; 22 Department of Psychiatry, Indiana University School of Medicine, Indianapolis, IN USA; 23 Department of Psychiatry, University of Iowa Carver College of Medicine, Iowa City, IA USA; 24 Department of Psychiatry, Washington University in St. Louis, St. Louis, MO USA; 25 University of California, San Diego, La Jolla, California, USA; 26 Department of Genetics, Rutgers University, Piscataway, NJ USA; 27 Department of Cell Biology and Neuroscience, Rutgers University, Piscataway, NJ USA; 28 Department of Genetics, Perelman School of Medicine, and the Penn-CHOP Lifespan Brain Institute, University of Pennsylvania, Philadelphia, PA USA; 29 Department of Genetics and Genomic Sciences, Ronald M. Loeb Center for Alzheimer’s Disease Icahn School of Medicine at Mount Sinai, New York, NY USA; 30 Nash Family Department of Neuroscience, Icahn School of Medicine at Mount Sinai, New York, NY USA; 31 Department of Pediatrics, Howard University, Washington, DC USA; 32 Department of Human Genetics, Howard University, Washington, DC USA; 33 Center of for Medical Genomics, Indiana University School of Medicine, Indianapolis, IN USA; 34 Department of Psychiatry, University of Iowa, Iowa City, IA USA; 35 NIAAA, Bethesda, USA

**Keywords:** Predictive markers, Genetics

## Abstract

Genome-wide association studies (GWAS) identify genetic variants associated with a trait, regardless of how those variants are associated with the outcome. Characterizing whether variants for psychiatric outcomes operate via specific versus general pathways provides more informative measures of genetic risk. In the current analysis, we used multivariate GWAS to tease apart variants associated with problematic alcohol use (ALCP-total) through either a shared risk for externalizing (EXT) or a problematic alcohol use-specific risk (ALCP-specific). SNPs associated with ALCP-specific were primarily related to alcohol metabolism. Genetic correlations showed ALCP-specific was predominantly associated with alcohol use and other forms of psychopathology, but not other forms of substance use. Polygenic scores for ALCP-total were associated with multiple forms of substance use, but polygenic scores for ALCP-specific were only associated with alcohol phenotypes. Polygenic scores for both ALCP-specific and EXT show different patterns of associations with alcohol misuse across development. Our results demonstrate that focusing on both shared and specific risk can better characterize pathways of risk for substance use disorders. Parsing risk pathways will become increasingly relevant as genetic information is incorporated into clinical practice.

## Introduction

Genome-wide association studies (GWAS) are rapidly advancing our ability to detect genetic loci associated with psychiatric disorders [1–6]. However, GWAS of any given outcome will detect genetic loci related to that outcome via correlated traits. This non-specificity is evident in the ubiquitous genetic correlations detected across psychiatric traits [1–6]. Using approaches that partition variance in traits can be useful in disentangling pleiotropic effects from those that are specific to a phenotype of interest. In the current analysis, we use the etiology of alcohol use disorders (AUD) as a primary example of how this approach can be useful.

AUDs are moderately heritable (~50%) [7], with most of the heritability of AUD shared with other externalizing phenotypes [8–10]. Externalizing generally refers to a broad liability towards behavioral disinhibition and dysregulation. The externalizing spectrum includes disorders such as attention-deficit/hyperactivity disorder (ADHD), conduct disorder, substance use disorders, and antisocial behavior personality disorder, as well as personality traits like impulsivity and sensation seeking [10–12]. Prior research estimates that the majority of the genetic variance for AUD is shared with other externalizing disorders with a much smaller proportion of genetic variation specific to alcohol use outcomes [8]. Additionally, genetic influences on alcohol use outcomes change over time, with externalizing liability being more important in adolescence and alcohol-specific risk becoming more important as individuals age [13, 14]. In total, research on the etiology of AUD suggests differing pathways through which problems can develop.

Herein, we apply new multivariate methods [15, 16] to tease apart specific versus shared pathways by which genetic loci are associated with problematic alcohol use and demonstrate how moving beyond GWAS that focus on a single outcome may help us better understand the manner in which risk for psychiatric problems unfolds. Specifically, we expand upon a recent multivariate GWAS of externalizing [17] to differentiate the genetic variants that impact problematic alcohol use through this broad externalizing liability (EXT), from variants that are specific to problematic alcohol use (ALCP-specific). We compare our multivariate results to those from a previously published meta-analysis of GWAS of problematic alcohol use [18, 19], which, by definition, combined all genetic pathways that impact risk for alcohol problems (ALCP-total). Across these three GWAS results, we compare: (1) the genetic correlations with other relevant phenotypes; (2) the biological annotations of each genetic signal, and (3) the associations of polygenic scores (PGS) for each component with a variety of substance use phenotypes. Our analyses aim to better characterize the genetic pathways associated with problematic alcohol use and illustrate the use of multivariate genomic analyses to differentiate patterns of risk.

## Methods

### GWAS of problematic alcohol use specific variance (ALCP-specific)

Our current analysis builds on results from a recently published multivariate GWAS of externalizing [17]. We used GenomicSEM [15] to fit a common factor model using summary statistics from seven externalizing-related phenotypes: attention-deficit/hyperactivity disorder (ADHD), problematic alcohol use (ALCP-total), lifetime cannabis use (CANN), age at first sexual intercourse (FSEX), number of sexual partners (NSEX), general risk tolerance (RISK), and lifetime smoking initiation (SMOK). This analysis suggested a single underlying latent genetic factor (EXT), with residual genetic variance on each phenotype. The original paper presented GWAS results for that latent factor EXT. Here, we extended the original model to partition SNP effects on ALCP-total into two pathways: those that are shared among externalizing phenotypes and those that are specific to problematic alcohol use (ALCP-specific) [17].

The details for how GWAS traits were selected are presented in detail in the original EXT analysis [17]. Briefly, we included GWAS of externalizing traits if studies had 50,000 or more participants, included relevant covariates (e.g., sex, age, ancestral principal components), were successfully genotyped genome-wide (individual genotyping rate: >95%), passed the standard quality controls, and were limited to unrelated individuals or used techniques to correct for relatedness. We standardized input summary statistics for each of the externalizing phenotypes and filtered to a set of common SNPs across each of the GWAS for a total of 6,132,068 common SNPs (MAF > 0.5%). Next, we performed association tests across all available SNPs, where EXT and ALCP-specific were sequentially regressed onto each SNP. All analyses were limited to samples of European ancestries. See Supplementary Note 2 for detailed information.

**Fig. 1:**
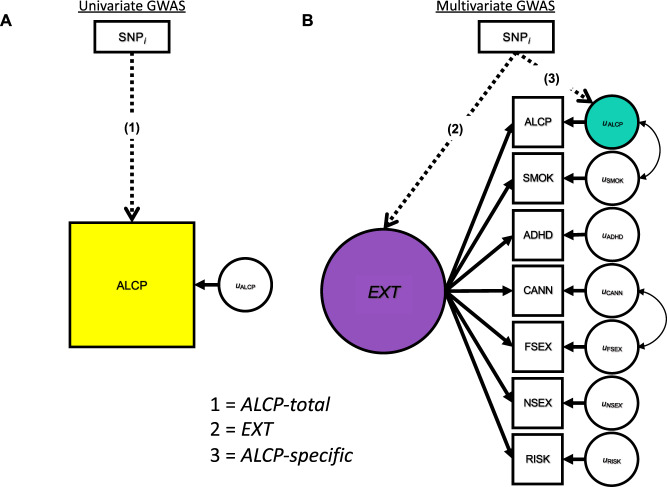
GWAS Models for Problematic Alcohol Use and Externalizing. Univariate (**A**) and multivariate (**B**) GWAS models for problematic alcohol use and externalizing. The SNP associations with the yellow box labeled ALCP are referred to herein as “ALCP-total” (original problematic alcohol use), the SNP associations with the purple circle are referred to herein as “EXT” (shared risk towards externalizing), and the SNP associations with the teal circle are referred to herein as “ALCP-specific” (problematic alcohol user-specific).

Figure 1 displays the three sets of GWAS results used to make comparisons. Figure 1A displays the univariate GWAS, focusing on a single phenotype (ALCP-total). Figure 1B displays the multivariate GWAS, with the seven indicators used to estimate the model. From these two models, we derive three sets of GWAS summary statistics: the univariate GWAS results for ALCP-total (path 1) [18, 19], the common factor GWAS results for EXT (path 2) [17], and the problematic alcohol use specific GWAS (path 3; ALCP-specific). These results allow us to dissect genetic variance in problematic alcohol use by comparing the results of ALCP-total to both EXT and ALCP-specific.

### Bioannotation

We compared biological annotations from each of the GWAS using a previously established pipeline [17]. First, we used FUMA [20] v1.2.8 to identify independent SNPs (LD threshold of *r*^2^ < 0.1) and conducted competitive gene-set, tissue and pathway analysis using MAGMA v1.08 [21]. Next, we used an extension of MAGMA, Hi-C coupled MAGMA (H-MAGMA) [22], to assign non-coding (intergenic and intronic) SNPs to genes based on their chromatin interactions. Lastly, we used S-PrediXcan v0.6.2 [23] to predict transcript abundance in 13 brain tissues, and to test whether the predicted transcripts showed divergent correlation patterns with each of the genetic factors. Supplementary Note 3 contains detailed information on the bioannotations.

### Genetic correlations

We estimated genetic correlations between EXT, ALCP-specific, ALCP-total and 99 preregistered phenotypes, again using GenomicSEM. A full list of the genetic correlations is available in Supplementary Table 2. Genetic correlations allowed us to examine how patterns of associations differed across EXT, ALCP-total, and ALCP-specific. In determining which genetic correlations to compare, we limited results to those which were significantly associated with ALCP-total after correcting for multiple testing [24].

### Polygenic scores

We created polygenic scores (PGS) from each set of GWAS summary statistics in particpants of primarily European ancestries from two independent cohorts: the National Longitudinal Study of Adolescent to Adult Health [25] (Add Health; *N* = 5107) and the Collaborative Study on the Genetics of Alcoholism [26] (COGA; *N* = 7594). Within each sample we created PGS for (1) ALCP-total; (2) EXT; and (3) ALCP-specific using PRS-CS, a Bayesian approach that uses a continuous shrinkage parameter to adjust GWAS summary statistics for linkage disequilibrium (LD) [27].

Within each of the holdout samples, we compared the effect size (*ΔR*^2^ above a model with covariates only) of the association of ALCP-total and ALCP-sp*e*cific PGS with five substance categories, including alcohol, cannabis, nicotine, opioids (COGA only), and other illicit substances (e.g., cocaine, sedatives, stimulants, methamphetamine). Outcomes included “ever use” and SUD criterion counts. All models included age, sex, the first ten ancestral principal components, and study-specific covariates.

Finally, we compared the association between the EXT and ALCP-specific PGSs with an alcohol use index (AUI) [28] across time using a linear growth model [29] in Add Health. This composite index included five alcohol phenotypes ranging from normative to problematic use, scaled to a value of 0 to 10 [28] and is well suited to capture the developmentally contingent definition of substance “misuse” (e.g., early life initiation, drinking to intoxication in adolescence, developing problems in adulthood). Detailed descriptions of the holdout samples, phenotypes, and analyses are presented in Supplementary Note 4.

## Results

### Lead SNPs and bioannotations

Table 1 presents lead SNPs from the ALCP-total GWAS across the three GWAS. In the ALCP-total GWAS, we identified 542 genome-wide significant (*p* < 5 × 10^–8^) SNPs before pruning for LD. Table 1 includes the 11 independent (LD threshold of *r*^2^ < 0.1) lead SNPs for ALCP-total, and their corresponding estimates in the EXT and ALCP-specific GWAS results. Of these, only the locus on chromosome 3 (rs10511087), in the *CADM2* region, was significant in the EXT GWAS, and the large number of SNPs before pruning were likely the result of a long-range LD region near *CADM2*. The lead SNP from another locus, located on chromosome 11, was in LD with one of the top SNPs from the EXT GWAS in *NCAM1* (rs9919558, *p* < 6.50 × 10^–59^). Notably, none of the SNPs on chromosome 4 were significant (or in LD) in the EXT GWAS. Instead, 8 of the 9 lead SNPs on chromosome 4 were significant in the ALCP-specific GWAS. The top SNPs for ALCP-specific are in *ADH1B* and *ADH1C*, which are involved in alcohol metabolism, as well as other genes previously associated with alcohol phenotypes including *KLB* [30].

**Table 1:** ALCP-total Lead SNPs across ALCP-total, EXT, and ALCP-specific GWASs.

				ALCP-total		EXT		ALCP-specific	
SNP	CHR	BP	Nearest Gene	Dir	−log10(P)	Dir	−log10(P)	Dir	−log10(P)
rs10511087	3	85439136	*CADM2*	+	**8.41**	**+**	**48.15**	+	3.26
rs6842066	4	39393801	*RNU6-887P*	**−**	**9.49**	+	0.94	**−**	**10.08**
rs28712821	4	39413780	*KLB*	**−**	**11.04**	+	0.81	**−**	**11.61**
rs1229984	4	100239319	*ADH1B*	**−**	**46.19**	+	1.66	**−**	**48.27**
rs3811802	4	100244221	*ADH1B*	**−**	**14.19**	+	1.06	**−**	**14.98**
rs3114045	4	100252560	*ADH1C*	**−**	**7.88**	+	0.67	**−**	**8.30**
rs4699743	4	100282103	*ADH1C*	+	**8.29**	−	0.57	**+**	**8.67**
rs111466094	4	100408974	*RP11-696N14.3*	+	**8.15**	−	2.06	**+**	**9.13**
rs13135092	4	103198082	*SLC39A8*	+	**14.51**	+	1.72	**+**	**13.23**
rs34333163	4	103283117	*SLC39A8*	+	**7.51**	+	1.37	+	6.72
rs35277073	11	113350620	*DRD2*	+	**7.49**	+	2.47	+	6.40

Independent lead SNPS from ALCP-total GWAS pruned for an LD threshold of *r*^2^ < 0.1.

Bolded = genome-wide significant (*p* < 5 × 10^−8^).

Gene-based analyses identified 13 genes associated with ALCP-total, but only 2 genes associated with ALCP-specific. Analysis of tissue expression in MAGMA and H-MAGMA did not allow for comparisons because of the limited power in the ALCP-specific results. In S-PrediXcan, only *ADH1C* was significantly associated with ALCP-specific. While these results do not point to any new biological pathways of risk, the biological sources of genetic variance across these different pathways reaffirm that EXT is capturing a broader risk domain, while ALCP-specific is identifying genes primarily associated with the pharmacokinetics of alcohol (see Supplementary Tables 8–11 for full results).

### Genetic correlations across ALCP-total and ALCP-specific

**Fig. 2:**
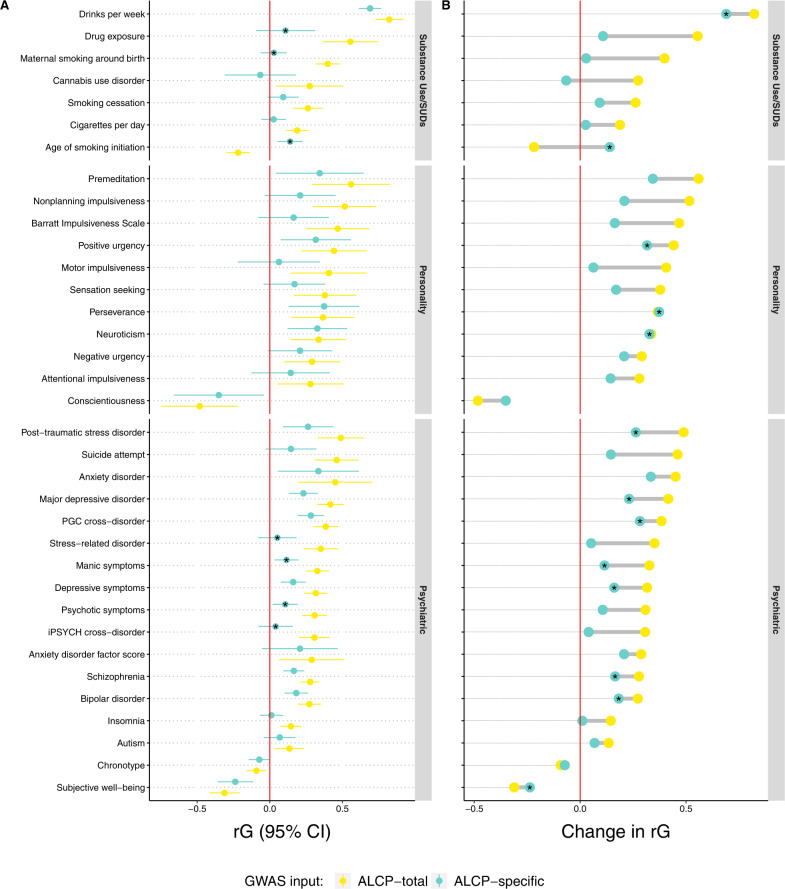
Values and differences in genetic correlations for ALCP-total and ALCP-specific. Panel (**A**) presents the genetic correlations (*r*_*g*_) and 95% CI between problematic alcohol use (ALCP-total, yellow dots), problematic alcohol use-specific (ALCP-specific, teal dots), and selected traits significantly correlated with ALCP-total (after correcting for an FDR of 5%, Supplementary Table 2 reports all preregistered genetic correlations). Asterisks (*) in Panel (**A**) represent genetic correlations for ALCP-specific that differ significantly from those with ALCP-total. Panel (**B**) presents changes in point estimates for genetic correlations from ALCP-total to ALCP-specific. In Panel (**B**), asterisks represent genetic correlations for ALCP-specific that are still significant (after correcting for an FDR of 5%).

ALCP-total was significantly correlated with 64 of the 99 preregistered phenotypes. We focus on the 35 traits related to substance use, personality, and other psychiatric outcomes (full results in Supplementary Table 2). Figure 2 provides two depictions of the results. Panel A presents the (*r*_*g*_) estimates (and 95% confidence intervals) between ALCP-total (yellow), ALCP-specific (teal), and the other traits. Here, the asterisks denote genetic correlations that differ significantly from ALCP-total to ALCP-specific. Three of the genetic correlations for substance related phenotypes (drug exposure, maternal smoking, and age of smoking initiation) and four genetic correlations for psychiatric traits (stress-related disorders, manic symptoms, psychotic symptoms, and iPSYCH cross-disorder) differed across the two sets of GWAS. None of the estimates for the personality related traits differed significantly across the sets of results.

Panel B presents the difference in the same genetic correlations, and the asterisks indicate a significant association with ALCP-specific after correcting for multiple testing. Overall, genetic correlations with other traits were attenuated for ALCP-specific compared to ALCP-total. For substance use, ALCP-total was genetically correlated with all other forms of substance use (*r*_*g*_ = −0.22–0.82). Once we remove the shared variance due to EXT, ALCP-specific was only associated with drinks per week (*r*_*g*_ = 0.69) and age of smoking initiation (*r*_*g*_ = 0.14). ALCP-total was genetically correlated with most of the impulsivity and personality phenotypes (*r*_*g*_ = −0.48–0.56). Only neuroticism (*r*_*g*_ = 0.33), lack of perseverance (*r*_*g*_ = 0.37), and positive urgency (*r*_*g*_ = 0.32) were correlated with ALCP-specific. Finally, ALCP-total was genetically correlated with various psychiatric traits (*r*_*g*_ = −31–0.49). ALCP-specific remained associated with most of these traits, notably bipolar disorder (*r*_*g*_ = 0.18), major depressive disorder (*r*_*g*_ = 0.23), post-traumatic stress disorder (*r*_*g*_ = 0.26), and schizophrenia (*r*_*g*_ = 0.17). Overall, a substantial portion of the observed genetic correlations between problematic alcohol use and other phenotypes is due to genetic variants that operate via externalizing liability.

### Polygenic scores and substance use disorders

**Fig. 3:**
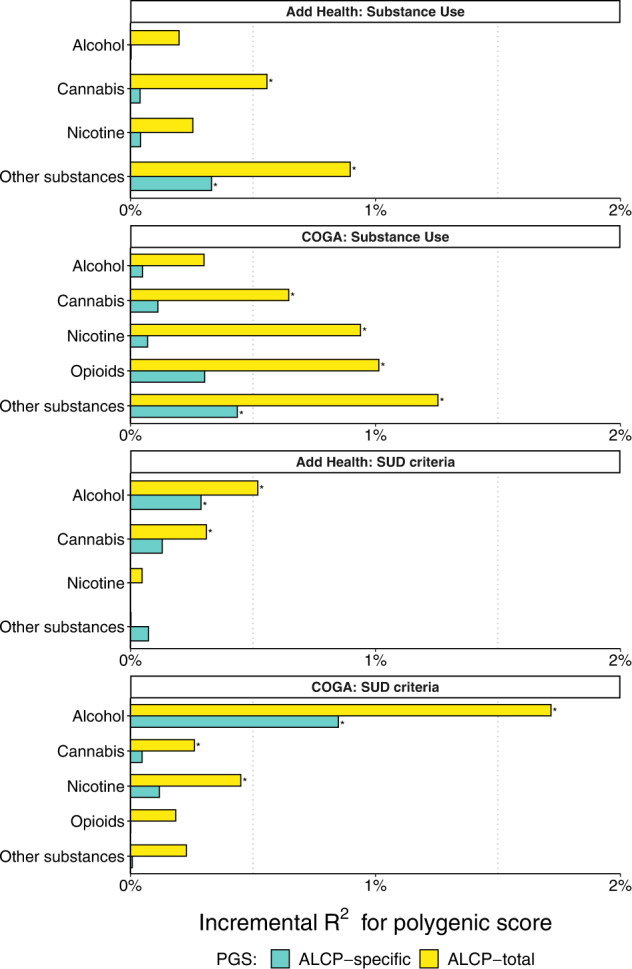
Polygenic associations with substance use and substance use disorders. Bar charts illustrating the incremental proportion of variance (incremental R2, or ΔR2 above model with age, sex, PCs, and study-specific covariates) explained by the polygenic score in Add Health (*N* = 5107) and COGA (*N* = 7594). Association between polygenic scores and lifetime SUD criterion counts for alcohol, cannabis, nicotine, other illicit substances, and opioids (COGA only). Asterisks (*) represent polygenic scores that are significant after correcting for an FDR of 5%.

Figure 3 illustrates associations between polygenic scores and various substance use phenotypes in the forms of *ever use* and *SUD criterion counts*. Neither the ALCP-total PGS nor the ALCP-specific PGS were associated with ever using alcohol in Add Health or COGA. For AUD criteria, the ALCP-total PGS explained 0.52% of the variance (Δ$R_{{\rm{ALCP}} {\mbox{-}} {\rm{total}}}^2$) in Add Health and 1.72% of the variance in COGA, compared to the ALCP-specific PGS, which explained 0.28% of the variance in Add Health and 0.85% of the variance in COGA. Supplementary Table 3 presents a full comparison of the EXT, ALCP-specific, and ALCP-total PGS for AUD criterion counts. The ALCP-total PGS was associated with cannabis use and other substance use in Add Health (OR_ALCP-total_ = 1.15–1.20, Δ*R*^2^ = 0.56–0.90%) and all forms of use in COGA (OR_ALCP-total_ = 1.20–1.27, Δ*R*^2^ = 0.65–1.26%). Additionally, the ALCP-total PGS was associated with cannabis use disorder criteria in both COGA and Add Health (*β*_ALCP-total_ = 0.11–0.16, Δ*R*^2^ = 0.26–0.31%), and nicotine dependence criteria in COGA (*β*_ALCP-total_ = 0.20, Δ*R*^2^ = 0.45%). However, the ALCP-specific PGS was only associated with ever using illicit substances (other than cannabis) and the associations are attenuated compared to that of ALCP-total (OR_ALCP-specific_ = 1.12–1.15, Δ*R*^2^ = 0.33–0.44%).

### Polygenic scores and longitudinal models of alcohol misuse

**Fig. 4:**
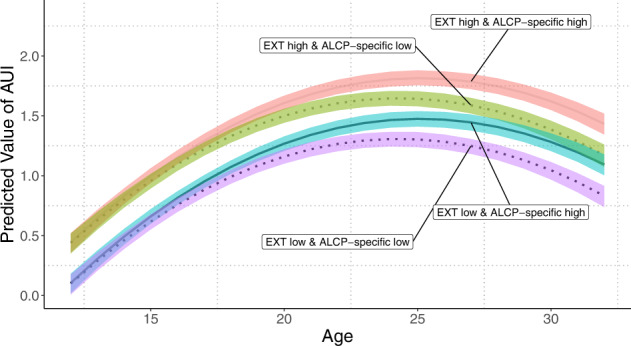
Longitudinal models of polygenic associations with alcohol use and misuse. Predicted values for alcohol use index (AUI) from ages 12 to 32 using linear mixed models in Add Health (*N* = 5107). Values for EXT and ALCP-specific polygenic scores set to ±1.5 SD. The shaded areas represent 95% confidence intervals. Confidence intervals estimated using percentile method bootstrapping over 1000 bootstrap samples. All other covariates set to mean values.

Lastly, we fit a series of longitudinal growth models for a composite alcohol use index (AUI) in Add Health (see Supplementary Note 4.5 for complete results). The best fitting model represented a quadratic change in AUI over time (with sex differences in slope), a significant association between EXT PGS and base levels of AUI (*β*_EXT_ = 0.12, SE_EXT_ = 0.01, *P*_EXT_ = 1.46 × 10^–18^), and a significant association between ALCP-specific PGS and change in the linear component of age for AUI (*β*_ALCP-specific*AGE_ = 0.07, SE_ALCP-specific*AGE_ = 0.02, *P*_ALCP-specific*AGE_ = 5.70 × 10^–5^). We found no evidence of sex-specific effects of either PGS in stratified models. Figure 4 provides a visual representation of the results from this best-fitting longitudinal model across levels of each PGS (±1.5 SD). Individuals higher on EXT PGS experience higher levels of AUI across time and sex, whereas those with higher levels of ALCP-specific experience increased growth in AUI. Supplemental Table 7 presents comparisons of estimates from longitudinal models using ALCP-total PGS to those in the main text.

## Discussion

Genetic influences on SUDs can operate via both shared genetic risk with other forms of externalizing as well as via substance specific pathways. Conventional analyses focused on a single phenotype, what we would term the “classical” GWAS approach, are unable to differentiate these pathways; newer multivariate approaches begin to make this possible. In the current analysis, we demonstrated the potential of disaggregating genetic variance in problematic alcohol use into risk shared with other externalizing behaviors/disorders versus risk that is specific to problematic alcohol use. We demonstrate that multivariate genomic analyses of correlated traits can increase the specificity for characterizing *how* genetic risk unfolds.

When we compared the results from the univariate ALCP-total GWAS to those from the multivariate model (EXT and ALCP-specific), we found robust evidence of distinct risk pathways. We compared the genetic correlations for ALCP-total and ALCP-specific across 99 preregistered phenotypes, focusing here on phenotypes related to personality, substance use, and psychopathology. ALCP-total was correlated with a broad range of personality phenotypes, especially those related to impulsivity. However, after removing the variance due to the shared risk for EXT, most of these associations were no longer significant. Similarly, ALCP-total was genetically correlated with multiple forms of other substance use phenotypes, while ALCP-specific remained correlated only with alcohol consumption and age of smoking initiation, and this latter correlation switched direction. The change in direction could reflect the fact that ALCP-specific captures (1) risk for problematic alcohol use once alcohol becomes available, or (2) risk for other psychiatric problems around their median age of onset, both of which occur during young adulthood [31], forcing the correlation with age of smoking initiation to be positive. It is also possible that this change in direction is merely a statistical artifact. Lastly, ALCP-total was correlated with a variety of psychiatric phenotypes. ALCP-specific continued to yield small associations with many psychiatric traits, suggesting that ALCP-specific contains signal related to other disorders. It is important to note that in addition to removing shared variance with EXT, some of these association may no longer be significant, in part, due to reduced statistical power in the ALCP-specific results.

At the SNP level, our approach was further able to separate alcohol-specific biology from a general risk towards externalizing, given that alcohol metabolizing genes (e.g., *ADH1B* and *ADH1C*) were significant in the alcohol-specific, but not EXT, results. We identified 11 lead SNPs after pruning for LD: one on chromosome 3, nine on chromosome 4, and one on chromosome 11. In the ALCP-specific GWAS, 8 of the 9 lead ALCP SNPs on chromosome 4 were genome-wide significant. These top SNPs were in alcohol metabolism genes (*ADH1B* and *ADH1C*), and other genes previously associated with alcohol phenotypes including *KLB* [30, 32]. Additionally, *SLC39A8* has been consistently identified as a risk variant for schizophrenia [5, 33, 34]. This association with *SLC39A8 could* indicate that the ALCP-specific contains variance that is not unique to alcohol (e.g., risk for internalizing or psychotic disorders). Two of these ALCP-total SNPs were in strong LD (*r*^2^ ~ 0.98) with top SNPs from the EXT results. The SNP on chromosome 3 was in LD with two SNPs in the *CADM2* region, while the SNP on chromosome 11 was in LD with a SNP on *NCAM1*. *CADM2* has been implicated in previous GWAS of other substance use phenotypes [35, 36], risky behaviors [36–38], and impulsivity [38]. Overall, the SNP level results broadly point to two distinct pathways of risk: one related to risk taking/impulsivity, and one specific to the body’s processing of alcohol, both of which are entwined in the univariate GWAS results for problematic alcohol use.

Finally, we evaluated polygenic scores in Add Health and COGA [14]. The PGS for ALCP-specific were almost exclusively related to alcohol phenotypes, indicating that the model successfully differentiates shared and specific risk. While there were differences in the magnitudes in effect sizes across COGA and Add Health, which could reflect differences in how the samples were ascertained (nationally representative sample vs clinically ascertained), the overall patterns were similar. In longitudinal models, EXT was associated with higher mean-levels of AUI while ALCP-specific was associated with increased growth in AUI. Notably, these results illustrate that externalizing genetic risk is associated with differences in AUI early in development. In contrast, during emerging adulthood, when alcohol use becomes legal and more readily accessible, there is further differentiation by alcohol-specific genetic risk. Therefore, alcohol-specific risk does not lead to alcohol problems without exposure to drinking, while broader externalizing risk captures propensity to drinking exposure across the life course. This longitudinal model reiterates the developmentally contextual nature of risk [13, 14]. Overall, the PGS results support the notion of a shared externalizing risk pathway and an alcohol-specific risk pathway.

Our analyses included several important limitations. First, they were limited to GWAS of European ancestries. Unfortunately, Genomic SEM requires larger sample sizes to obtain stable estimates. As larger sample sizes become available in non-European ancestries, we will extend these models to those populations. Genetic research in diverse ancestries is important scientifically, but also morally, as failure to diversify genetic discovery will result in the exacerbation of health disparities [39]. Second, while we considered externalizing phenotypes, we did not consider internalizing or psychotic conditions, which also show genetic overlap with AUD and other substance use disorders [4, 6, 18, 40, 41]. Finally, our estimates of SNPs associated with ALCP-specific were limited by the relatively small discovery sample size for ALCP-total (*N* ~ 150 K). We do note that our GWAS of ALCP-total was highly correlated with the largest meta-analysis of problematic alcohol use to date (rG = 0.94, *p* = 4.94 × 10^–324^) [3]. Future iterations with more powerful GWAS of problematic alcohol use may reveal additional variants associated specifically with ALCP. Additionally, including traits related to alcohol-specific biological processes may further help distinguish alcohol-specific from other processes through which AUD develops.

GWAS of psychiatric disorders contain a mixture of different signals. Moving beyond univariate to multivariate GWAS designs offers the potential to tease apart these signals. Herein, we decomposed the genetic variation of problematic alcohol use into that which is shared with other externalizing phenotypes from that which is specific to problematic alcohol use. Comparison of results at multiple levels showed that variance specific to problematic alcohol use was related to alcohol phenotypes while that which was shared was more strongly related to other forms of substance use and impulsivity. Differentiating these pathways of risk will become more important as genetic data becomes incorporated into clinical practice.

## Supplementary information

Supplmental information

Supplmental tables

## Data Availability

All data sources are described in the manuscript and supplemental information. No new data were collected. Only data from existing studies or study cohorts were analyzed, some of which have restricted access to protect the privacy of the study participants. The GWAS summary statistics for the EXT GWAS, can be obtained by following the procedures detailed at https://externalizing.org/request-data/. Summary statistics are derived from analyses based in part on 23andMe data, for which we are restricted to only publicly available report results for up to 10,000 SNPs. The full set of externalizing GWAS summary statistics can be made available to qualified investigators who enter into an agreement with 23andMe that protects participant confidentiality. Once the request has been approved by 23andMe, a representative of the Externalizing Consortium can share the full GWAS summary statistics. Access to genetic data for COGA (dbGaP Study Accession: phs000763.v1.p1) and Add Health (dbGaP Study Accession: phs001367.v1.p1) are available through dbGaP.

## Code availability

No custom algorithms or software was developed in this study. All code is available by request from the corresponding author.
