# Supplementary material for: Parsing genetically influenced risk pathways: genetic loci impact problematic alcohol use via externalizing and specific risk
Source: Transl Psychiatry. 2022 Sep 30;12:420. doi: 10.1038/s41398-022-02171-x (PMC9525649; doi:10.1038/s41398-022-02171-x)
Supplement: Supplementary file 1 — Supplmental information [file 41398_2022_2171_MOESM1_ESM.docx]

Supplementary Information

**Parsing Genetically Influenced Pathways: Genetic Loci Impact Problematic Alcohol Use Via Externalizing and Specific Risk**

Correspondence to: [peter.barr@downstate.edu](mailto:ddick@vcu.edu), [danielle.m.dick@rutgers.edu](mailto:danielle.m.dick@rutgers.edu)

Table of contents

[Supplementary Information 1](#_Toc107431251)

[1 Study introduction 4](#_Toc107431252)

[2 GWAS 5](#_Toc107431253)

[2.1 Methods 5](#_Toc107431254)

[2.1.1 Phenotype definition 5](#_Toc107431255)

[2.1.2 Univariate genome-wide association analyses 5](#_Toc107431256)

[2.1.3 Multivariate genome-wide association analyses 5](#_Toc107431257)

[2.2 Results 6](#_Toc107431258)

[2.3 Genetic Correlations 6](#_Toc107431259)

[3 Bioannotation 8](#_Toc107431260)

[3.1 Methods 8](#_Toc107431261)

[3.1.1 Functional mapping and annotation with FUMA 8](#_Toc107431262)

[3.1.2 Gene-based, gene-set, and gene-property analyses with MAGMA 8](#_Toc107431263)

[3.1.3 Gene-based association using transcriptomic data with S-PrediXcan 9](#_Toc107431264)

[3.2 Results 9](#_Toc107431265)

[3.2.1 Results from the functional mapping and annotation with FUMA 9](#_Toc107431266)

[3.2.2 Results from the analyses with MAGMA, H-MAGMA, and S-PrediXcan 9](#_Toc107431267)

[4 Polygenic Scores 13](#_Toc107431268)

[4.1 Samples 13](#_Toc107431269)

[4.1.1 The National Longitudinal Study of Adolescent to Adult Health (Add Health) 13](#_Toc107431270)

[4.1.2 The Collaborative Study on the Genetics of Alcoholism (COGA) 13](#_Toc107431271)

[4.2 Polygenic Score Creation 13](#_Toc107431272)

[4.2.1 Adjustment of GWAS effect sizes for linkage disequilibrium (LD) 13](#_Toc107431273)

[4.2.2 Polygenic scores 14](#_Toc107431274)

[4.2.3 Substance Use/Substance Use Disorder (SUD) Analyses 14](#_Toc107431275)

[4.2.4 Longitudinal Growth Models 15](#_Toc107431276)

[4.3 Phenotype definitions 16](#_Toc107431277)

[4.3.1 Substance use 16](#_Toc107431278)

[4.3.2 Substance use disorders 16](#_Toc107431279)

[4.3.3 Alcohol Use Index (AUI) 17](#_Toc107431280)

[4.4 Results of SUD analyses in Add Health and COGA 18](#_Toc107431281)

[4.4.1 Ever Substance Use 18](#_Toc107431282)

[4.4.1 Substance Use Disorder Criterion Count 19](#_Toc107431283)

[4.5 Results of Longitudinal Growth Models in Add Health 19](#_Toc107431284)

[4.5.1 Initial model building 19](#_Toc107431285)

[4.5.2 Longitudinal Models of Polygenic Risk and Alcohol Misuse 20](#_Toc107431286)

[5 References 23](#_Toc107431287)

# Study introduction

Psychiatric disorders rarely occur in isolation, as evidenced by substantial cooccurrence among many psychiatric conditions. In addition to co-occurrence at the phenotypic level, there is also evidence of widespread genetic overlap across these disorders. Virtually all psychiatric disorders, including mood disorders ^1–3^, anxiety disorders ^4,5^, substance use disorders ^6–8^, and other forms of severe mental illness ^9^ are correlated to some degree at the genetic level ^10^.

While informative, these genetic correlations do not reveal the pathways through which risk for certain disorders unfolds. Individual genome wide association study (GWAS) signals contain a mixture of variants that influence the outcome through different pathways of risk. Here we highlight how new multivariate genomic methods can help us tease apart these pathways of risk, using the overlap between problematic alcohol use and other externalizing problems as an example.

Twin and family studies estimate the heritability of alcohol use disorder (AUD) to be approximately 50% ^11^. A large portion (~65 - 80%) of the heritability in AUD is shared with other psychiatric and behavioral phenotypes related to externalizing ^12,13^. Externalizing refers to a general liability towards behavioral disinhibition and dysregulation. Externalizing includes disorders such as attention-deficit/hyperactivity disorder (ADHD) and conduct disorder in youth ^14,15^, substance use and antisocial behavior in adults ^16,17^, and normal range personality traits, such as risk tolerance, and sensation seeking ^18,19^. Multiple studies have examined the phenotypic ^16,19–22^ and etiological ^12,13,17,18,23–27^ overlap between externalizing traits.

In the current analysis, we conduct a multivariate GWAS to capture genetic variance specific to problem alcohol use and demonstrate the utility in identifying genetic variance unique to a trait of interest. We do so by building upon a prior multivariate GWAS of externalizing phenotypes ^27^, composed of (1) attention-deficit/hyperactivity disorder (ADHD), (2) problem alcohol use (ALCP), (3) lifetime cannabis use (CANN), (4) age at first sexual intercourse (FSEX), (5) number of sexual partners (NSEX), (6) general risk tolerance (RISK), and (7) lifetime smoking initiation (SMOK). GWAS of residual phenotypes capture only a portion of the total genetic architecture of a target trait, resulting in a more specific signal. This allows for direct tests of pleiotropy, identification of specific associated variants and biological processes leading to disease, and comparison of the influence of general and specific risk pathways.

# GWAS

In this section, we briefly describe the methods used to conduct the genome-wide association analyses reported in the present paper – with an emphasis on the ALCP-total (original, or univariate) and ALCP-specific (specific, or residual from multivariate) phenotypes. A thorough description of these genome-wide association analyses can also be found in our previously published study ^27^.

## Methods

### Phenotype definition

In the present study, we sought to examine the general and specific genetic influences on ‘problematic alcohol use’. We operationalized this phenotype as a combination of two GWASs: (1) a dimensional measure of problems related to alcohol consumption, and (2) a categorical measure of alcohol dependence. The former, a GWAS of the Problems subscale of the Alcohol Use Disorder Identification Test (AUDIT-P) ^28^, was analyzed in a subset of the UK Biobank (UKB) (*N* = 130,999) ^29^. The latter, a GWAS of alcohol dependence, was conducted by the Psychiatric Genomics Consortium (PGC) (*N* = 33,685 after excluding our follow-up study cohorts) ^6^. Given the large genetic correlation between these two phenotypes (*r*_g_ = 0.79), results were meta-analyzed to generate a single GWAS of problematic alcohol use.

### Univariate genome-wide association analyses

We used BOLT-LMM ^30,31^ v2.3.2 to conduct univariate genome-wide association analyses of AUDIT-P in UKB. As BOLT-LMM accounts for genetic relatedness among participants via a genetic variance component (pseudo *h*^2^), its linear mixed model approach offers increased statistical power via the inclusion of related participants. Here, we estimated the genetic variance component using a set of 483,680 typed autosomal single-nucleotide polymorphisms (SNPs) that (i) passed the quality control procedures employed by the original UKB investigators^32^, (ii) had a minor allele frequency (MAF) greater than 0.005, (iii) had a Hardy-Weinberg-Equilibrium (HWE) *P* value greater than 1×10^–16^, and light LD-pruning (window size = 50 kb; variant step-size = 5; *r*^2^ ≥ 0.9). Participants were excluded from analyses if they (i) did not pass the sample-level quality control thresholds employed by the original UKB investigators^32^; (ii) were part of the UKB holdout sample (or were related to those participants as indicated by a pairwise KING coefficient ≥ 0.0442); (iii) did not self-report their ethnicity as “White”, “White British”, “White Irish”, or “Any other white background”; (iv) had discordant self-reported and genetic sex; (v) had putative sex chromosome aneuploidy; or (vi) had missing data with respect to the outcome or covariates.

We analyzed the third release of the UKB imputed genotype data. As previously reported, we included the following variables as covariates in the association analyses: sex, birth year, sex-by-birth year interactions, genotyping batch, and the first 40 genetic principal components of ancestry (see Linnér et al. 2021 ^27^ for further detail). GWAS summary statistics were subsequently subjected to quality control procedures as previously described ^27^. Finally, we used METAL ^33^ to conduct a sample-size-weighted meta-analysis of our AUDIT-P GWAS and the PGC’s alcohol dependence GWAS.

### Multivariate genome-wide association analyses

As we have previously described, we used Genomic Structural Equation Modeling (Genomic SEM) ^34^ to analyze the joint genetic architecture of externalizing phenotypes (see Linnér et al. 2021 ^27^ for additional detail). The final confirmatory factor model included summary statistics from seven externalizing phenotypes: ADHD, ALCP, CANN, FSEX, NSEX, RISK, and SMOK. After identifying the best-fitting model (Figure 1B), we used the GenomicSEM v0.0.2 package in R to conduct multivariate genome-wide association analyses of the latent genetic externalizing factor (EXT) and the residual genetic variance in ALCP (ALCP-specific).

We first used the *sumstats* function to standardize the univariate GWAS summary statistics for each of the externalizing phenotypes. After excluding SNPs that were not present across all indicators, we retained a total of 6,132,068 SNPs for analysis at this stage. We then used the *userGWAS* function to perform the association test in an iterative manner for each SNP, where EXT and ALCP were both regressed onto the target SNP. These analyses were performed for SNPs that were available in all univariate summary statistics, had a minor allele frequency ≥ 0.5%, and were present in the 1000 Genomes Phase 3 (version 5) reference panel. As previously reported, we specified unit loading identification in these Genomic SEM models with SNP effects, where the factor loading of NSEX was fixed to 1 to set the scale of EXT. The field-standard threshold of *P* < 5×10^–8^ was used to designate genome-wide significance.

## Results

The results of the genome-wide association analyses for ALCP-total and ALCP-specific are presented in **Supplementary** **Figures 1** and **2**, as Manhattan plots and quantile-quantile (Q-Q) plots, respectively. Briefly, we found robust polygenic signals for both ALCP-total and ALCP-specific, as evidenced by substantial inflation of the test statistics ($\lambda_{\mathrm{GC}}=$ 1.162 and $\lambda_{\mathrm{GC}}=$ 1.102, respectively), LD Score regression intercepts (intercept = 1.013 and intercept = 0.990, respectively), and attenuation ratios (ratio = 0.068 and ratio = 0.000, respectively) for both phenotypes. The SNP-based heritability was *h^2^_snp_* = 0.055 (SE = .004, *p* = 1.36*10^-39^) for ALCP-total and *h^2^_snp_* = 0.041 (SE = .004, *p* = 1.70*10^-25^) for ALCP-specific. Considered together, the observed inflation in test statistics appears to be due to a polygenic architecture rather than confounding or bias.

Before pruning for LD, we found that 542 SNPs were associated with ALCP-total at the level of genome-wide significance (*P* < 5x10^-8^). As noted in the main text, 465/542 of these SNPs were genome-wide significant in EXT (~86%) and 60/542 were genome-wide significant in ALCP-specific (~11%). There was no overlap between these two sets. Examination of the lead SNPs for ALCP-total in the EXT and ALCP-specific results further emphasizes that our ‘residual GWAS’ framework was successful in delineating general versus specific genetic effects on problematic alcohol use (**Supplementary** **Table 1**).

## Genetic Correlations

We also used Genomic SEM to estimate genetic correlations between the latent genetic externalizing factor and complex traits that were broadly related to four domains: demography, health and medicine, psychopathology, and socioeconomic outcomes. We used a standard Benjamini-Hochberg false discovery rate correction (FDR 5%) ^35^ to account for multiple testing. Results for all phenotypes are presented in **Supplementary** **Table 2.**

Supplementary Figure 1: Manhattan Plot and QQ-plot for ALCP-total GWAS Results

Supplementary Figure 2: Manhattan Plot and QQ-plot for ALCP-specific GWAS Results

# Bioannotation

In this section, we describe analyses comparing the biological function of results from the ALCP-total and ALCP-specific GWASs, by applying several functional genomics tools to annotate and prioritize putative regulatory variants. These methods include functional annotations (i.e., CADD scores to identify highly deleterious SNPs), mapping annotations (i.e., eQTL SNP-gene expression association); as well as gene- and transcriptome-based analyses (MAGMA, H-MAGMA, and S-PrediXcan). Details of each method are presented below, and the results are reported in **Supplementary Tables 7-10**.

## Methods

### Functional mapping and annotation with FUMA

We used Functional Mapping and Annotation of genetic associations (FUMA v1.3.5e)^36^ to study the functional consequences of the lead SNPs from the ALCP-total and ALCP-specific GWASs, reported in **Supplementary Table 1**, which included ANNOVAR categories (*i.e.*, the functional consequence of SNPs on genes), Combined Annotation Dependent Depletion (CADD) scores (*i.e.*, a measure of how deleterious a SNP is; greater than 12.37 is the suggested threshold to classify a SNP as deleterious), RegulomeDB scores (*i.e.*, a categorical score from 1a to 7 with 1a corresponding to the most biological evidence that the SNP is a regulatory element). The sources of the external reference data used in these analyses are fully described elsewhere^36^.

### Gene-based, gene-set, and gene-property analyses with MAGMA

We performed competitive gene-based association analyses using the genome-wide summary statistics from each GWAS by applying the method “multi-marker analysis of genomic annotation” (MAGMA v1.08) ^36,37^. First, we assigned SNPs to genes based on physical position (gene-based analysis). SNPs were mapped to 18,318 protein-coding genes from Ensembl build 85. This approach uses multiple regression methods to account for LD between SNPs. All variants within all protein-coding genes were tested, using default settings, with LD structure estimated using the 1000 Genomes European sample as a reference. We evaluated Bonferroni-corrected significance, adjusted for testing 18,318 genes (one-sided *P* < 2.73×10^–6^). The results are reported in **Supplementary Table 8**.

Next, we performed a MAGMA gene-set analysis (the results are reported in **Supplementary Table 9**). We used 15,496 curated gene sets and Gene Ontology (GO) terms obtained from the Molecular Signatures Database (MsigDB v7.0, <https://www.gsea-msigdb.org/gsea/msigdb/index.jsp>) ^38^, which characterize the biological processes, molecular function and cellular component of individual gene products. We evaluated Bonferroni-corrected significance, adjusted for testing 15,496 gene sets (one-sided *P* < 3.23×10^–6^).

Lastly, we performed a gene property analysis to test the relationships between 54 tissue-specific gene expression profiles and gene associations (the results are reported in **Supplementary Figures 3-4**). We performed this analysis using the average expression of genes per tissue type as a gene covariate. Gene expression values were log_2_ transformed average RPKM (Reads Per Kilobase Million) per tissue type (after replacing RPKM > 50 with 50) based on GTEx RNA-seq data. We applied Bonferroni correction (one-sided *P* < 9.26×10^–4^) to correct for testing 54 gene expression profiles.

We used an extension of MAGMA v1.8, “Hi-C coupled MAGMA” or “H-MAGMA” ^39^, to assign non-coding (intergenic and intronic) SNPs to cognate genes based on their chromatin interactions. Exonic and promoter SNPs were assigned to genes based on physical position. We used four Hi-C datasets derived from adult brain ^40^, fetal brain ^41^, and iPSC derived neurons and astrocytes ^42^ (all available for download: <https://github.com/thewonlab/H-MAGMA>). The results are reported in **Supplementary Table 10**. We evaluated Bonferroni corrected *P*-value thresholds, adjusted for multiple testing within each analysis (one-sided *P* < 9.84×10^–7^, *P* < 9.86×10^–7^, *P* < 9.84×10^–7^, and *P* < 9.83×10^–7^, respectively).

### Gene-based association using transcriptomic data with S-PrediXcan

We used S-PrediXcan v0.6.2 ^43^ to analyze gene expression levels in multiple brain tissues, and to test whether the gene expression correlated with either ALCP-total or ALCP-specific. The results are reported in **Supplementary Table 11**. We used pre-computed tissue weights from the Genotype-Tissue Expression (GTEx, v8) project database (<https://www.gtexportal.org/>) as the reference transcriptome dataset ^44^. As input data, we used the summary statistics from each GWAS, transcriptome tissue data, and covariance matrices of the SNPs within each gene model (based on HapMap SNP set; available to download at the PredictDB Data Repository, <http://predictdb.org>) from 13 brain tissues: anterior cingulate cortex, amygdala, caudate basal ganglia, cerebellar hemisphere, cerebellum, cortex, frontal cortex, hippocampus, hypothalamus, nucleus accumbens basal ganglia, putamen basal ganglia, spinal cord and substantia nigra. We used a transcriptome-wide significance threshold of *P* < 2.72×10^–7^, which is the Bonferroni-corrected threshold when adjusting for 13 tissues times 14,117 tested genes (183,521 gene-tissue pairs).

## Results

### Results from the functional mapping and annotation with FUMA

11 SNPs were genome-wide significantly associated with ALCP-total. The loci were located in 5 genomic regions spanning across 3 chromosomes, including the genes *CADM2*, *RNU6-887P*, *KLB*, *ADH1B*, *ADH1C*, *RP11-696N14.3*, *SLCC9A8*, *DRD2*. The SNPs spanning in these regions were primarily intronic (77.9%), 16.3% being intergenic and only 0.07% being exonic. On the other hand, 9 SNPs were genome-wide significantly associated with ALCP-specific. All the loci were located on chromosome 4, and included the same genes identified in ALCP-total in this region (*KLB*, *ADH1B*, *ADH1C*, *RP11-696N14.3*, *SLCC9A8*). The majority of the SNPs identified with ALCP-specific were intergenic (43%) or intronic (41%), a greater majority of the SNPs (2.7%) being exonic as compared to ALCP-total. Notably, the genes *CADM2* and *DRD2* were only implicated with ALCP-total but not ALCP-specific.

### Results from the analyses with MAGMA, H-MAGMA, and S-PrediXcan

In the MAGMA analysis, a total of 13 genes were found associated with ALCP-total at a Bonferroni-corrected significance (one-sided P < 2.73×10^–6^), including several novel genes that were not identified via GWAS, such as *MTCH2* (previously implicated with body mass index and other metabolic phenotypes ^45,46^), *CELF1* (also previously implicated with body mass index ^47^), *FNBP4* (previously associated with neuroticism ^48^ and nervousness ^49^), among others. On the contrary, only *KLB* and *ADH1B* were associated with ALCP-specific in this analysis. Full results are presented in **Supplementary Table 10**.

Tissue enrichment analyses for both ALCP-total and ALCP-specific (**Supplementary Figures 3-4**) did not reach Bonferroni correction.


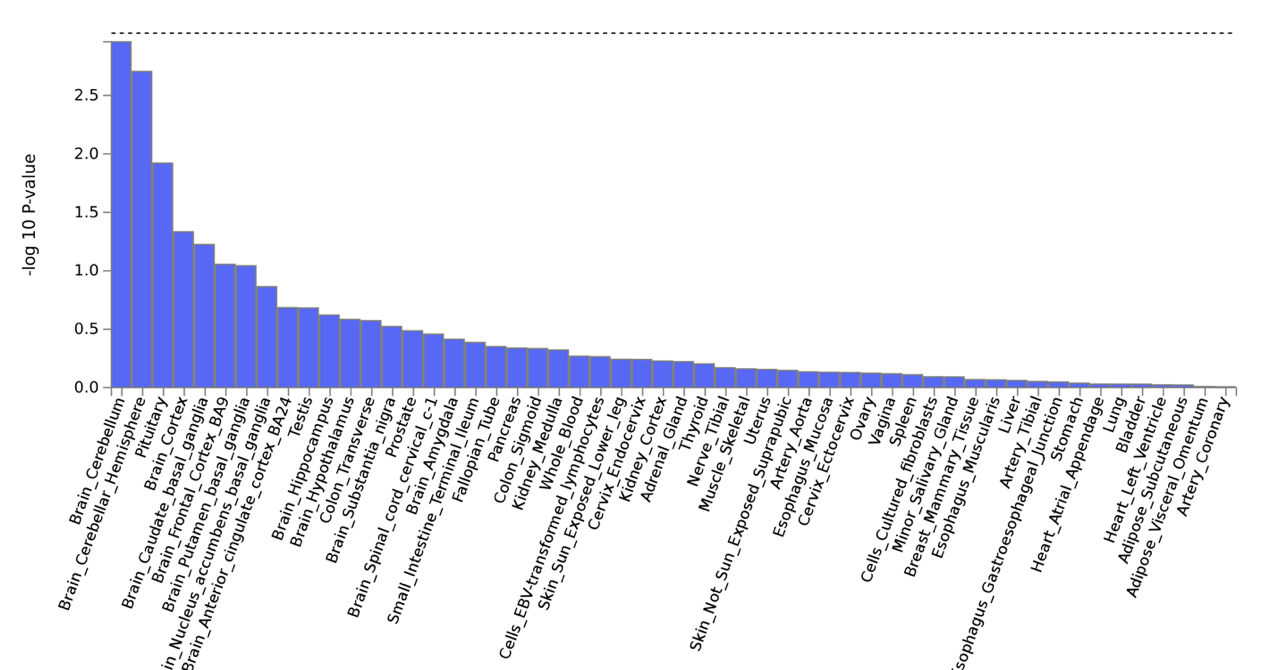

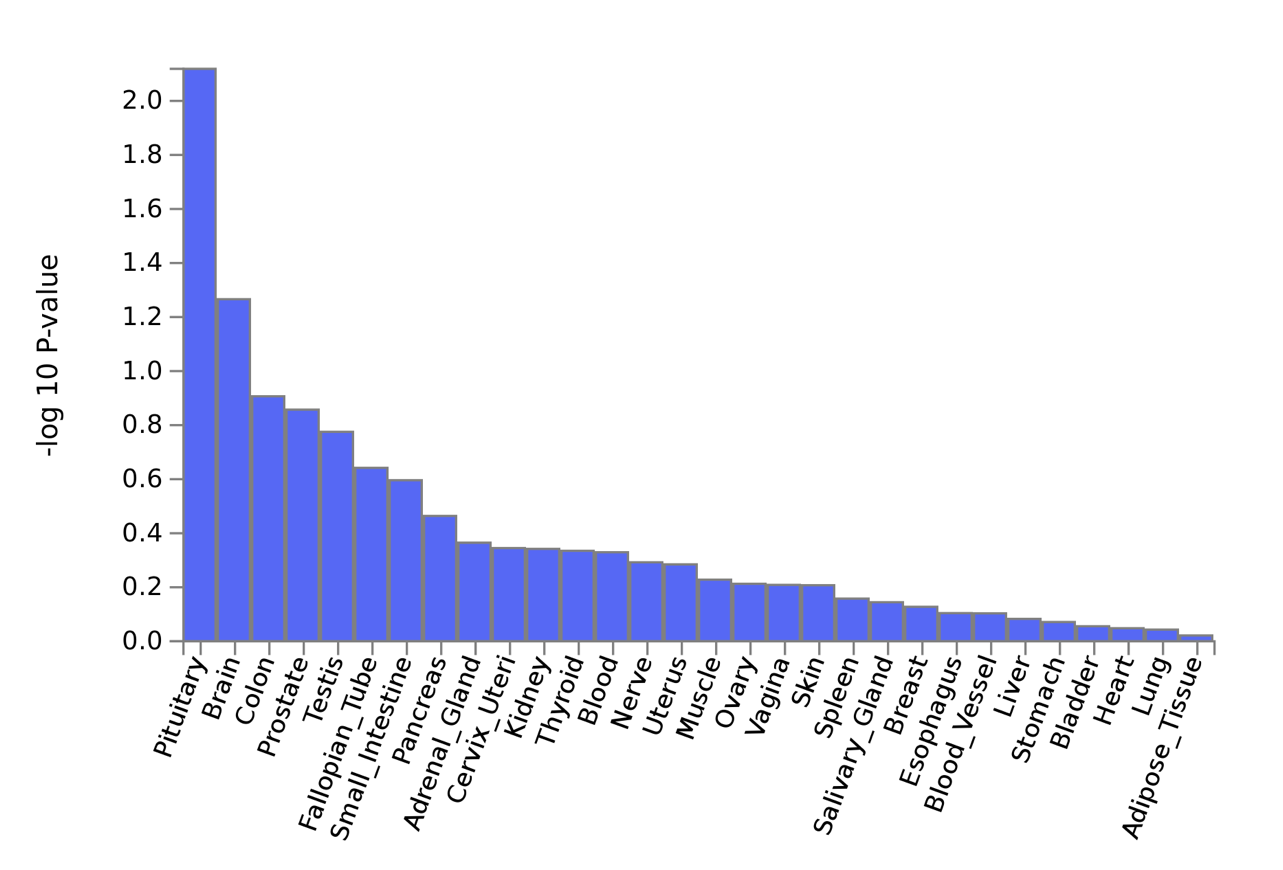


Supplementary Figure 3: Gene property analyses results for ALCP-total


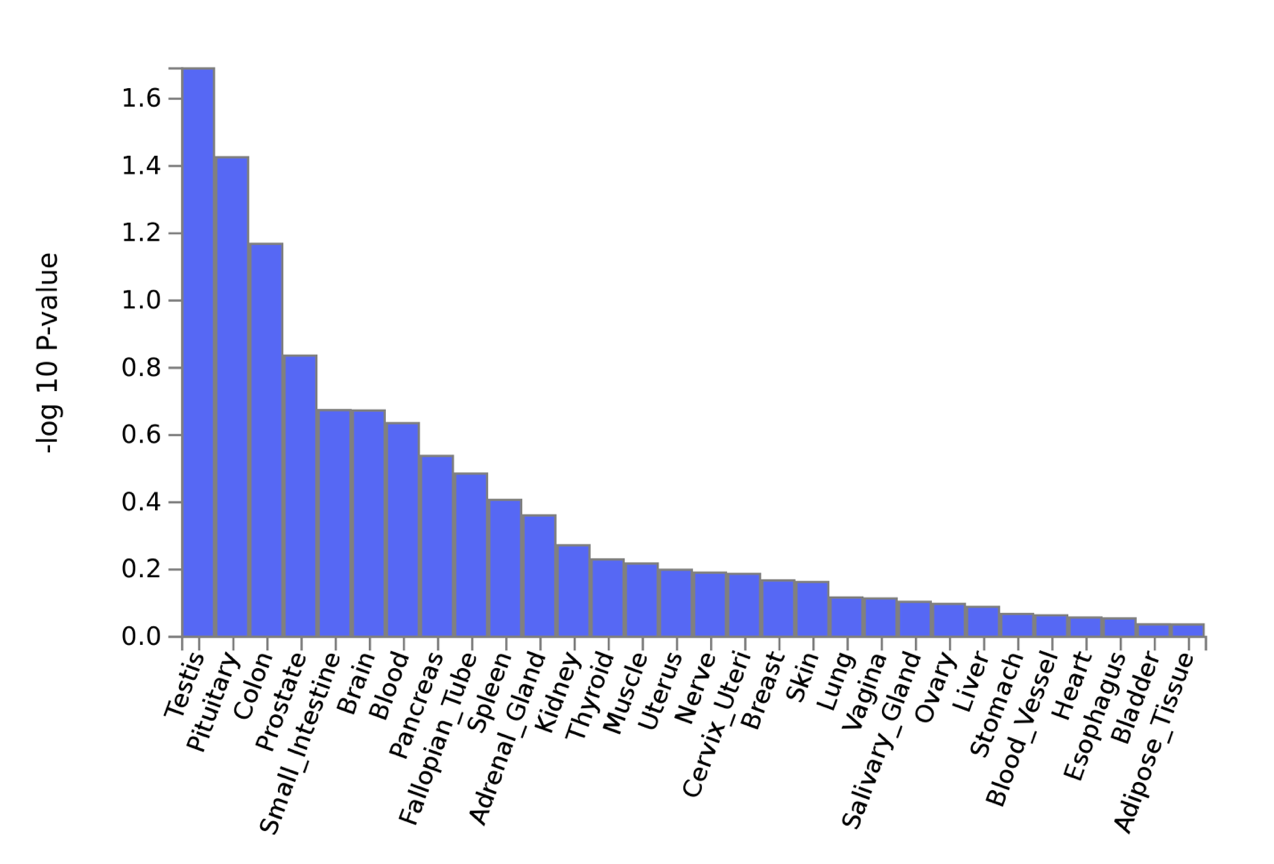

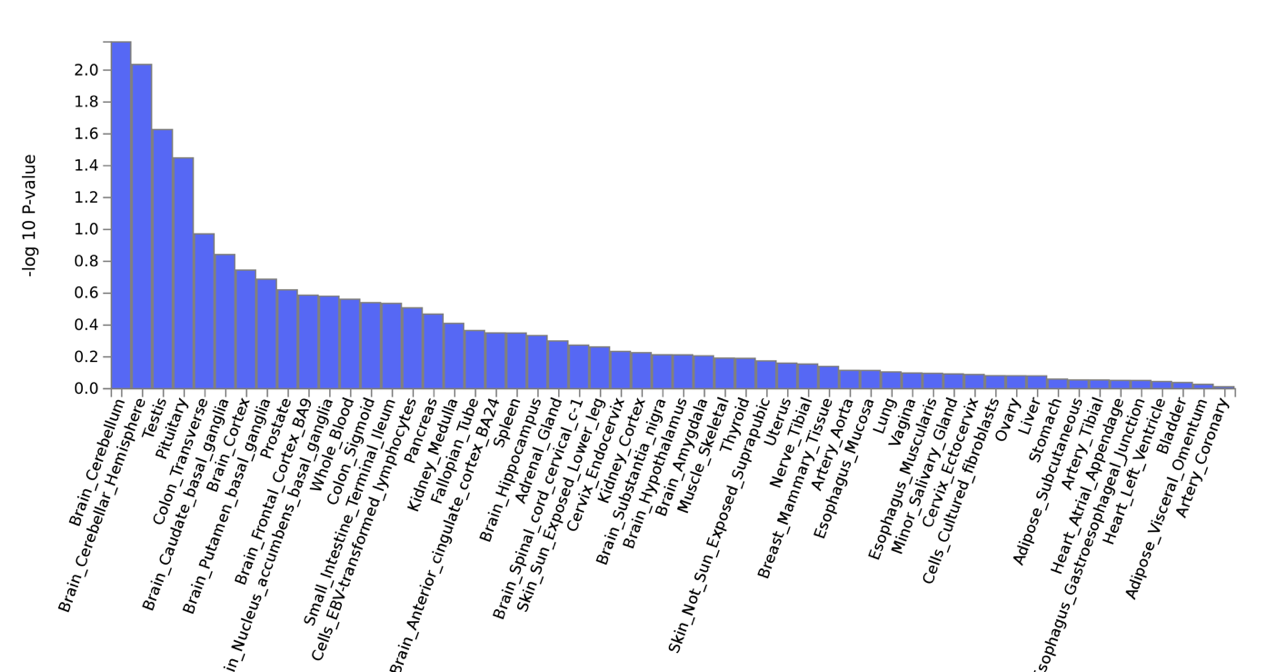


Supplementary Figure 4: Gene property analyses results for ALCP-specific

Next, by analyzing gene regulatory relationships using H-MAGMA (**Supplementary Table 10**), we identified significant associations with ALCP-total in adult brain tissue (19 genes), fetal brain tissue (17 genes), iPSC-derived astrocytes (14 genes), and iPSC-derived neurons (17 genes). Of these 33 unique genes, the majority of the genes were also implicated in MAGMA gene-based analyses, including *DRD2*, *ADH1B*, *CELF1*, *FNBP4*, *MTCH2*, among others. In addition, H-MAGMA analyses implicated other ethanol metabolizing genes (*ADH4*, *ADH1A*), as well as other genes previously identified with alcohol, such as *TSPAN5* ^18^ or SLC39A13 ^50^. H-MAGMA also implicated other novel genes, such as *MIR5591*. Of these 33 genes, 29 were also implicated in the H-MAGMA analyses for ALCP-specific, including *CADM2* or *DRD2*, which were not genome-wide significant in the GWAS, nor they were implicated in the MAGMA analysis. H-MAGMA analyses also revealed additional novel genes for ALCP-specific, including WDR19 (previously nominally associated with cognitive function ^51^).

Transcriptome-wide analyses with S-PrediXcan implicated *ADH1C* to be downregulated in the putamen (basal ganglia) both for ALCP-total and ALCP-specific (**Supplementary Table 11**). S-PrediXcan analysis also identified and association between *NDUFS3* and ALCP-total, low cerebellar expression of *NDUFS3* being associated with ALCP-total (but not ALCP-specific). *NDUFS3* has been previously associated with several other traits, including body size, insomnia, brain volume, loneliness, and cognitive performance ^52–58^.

# Polygenic Scores

In this section, we report on the construction and evaluation of polygenic scores (PGS) which we computed with weights from the problem alcohol use (ALCP), externalizing (EXT), and alcohol-specific (ALCP-specific) GWASs. We compare the relative strength of each PGS using the incremental *R^2^* attained by adding the polygenic score to a regression model with baseline covariates, in accordance with previous efforts ^18,27^. These analyses can be used to further refine pathways of risk for alcohol and other substance use disorders. Holdout Samples

## Samples

### The National Longitudinal Study of Adolescent to Adult Health (Add Health)

Add Health is *nationally representative sample* with five waves of data collection spanning adolescence (ages 11-18 at baseline) through early midlife (ages 32-42 in the current follow-up). Add Health participants were selected from a stratified sample of 132 schools resulting in an initial, nationally representative sample of 90,118 students in grades 7-12. Of the original sample, 20,745 were selected for additional in-home interviews. Of those who completed the Wave I interview (1994-1995), 14,738 (71%) completed Wave II (1996); 15,197 (73%) completed Wave III (2001-2002); and 15,701 (75%) completed Wave IV (2007-2008). Most respondents completed the majority of the waves, with 16,278 (78%) completing three or more waves. Wave V (ages 32-42) data collection is underway, with a target sample of 19,828 (data for N=3872 is already released). In total, 15,159 individuals interviewed during Wave IV (ages 24-32) provided samples for genotyping, conducted using the Illumina Omni1 and Omni2.5 arrays. After quality control, genotypic data are available for 9,974 individuals (5,896 non-Hispanic White; 2,081 African American; 1,448 Hispanic; 550 Other) ^59^.

### The Collaborative Study on the Genetics of Alcoholism (COGA)

COGA, initiated in 1989 to identify vulnerability genes for AUD, ascertained *high-risk families* through adult probands in treatment for AUD. In the first 10 years, probands along with all willing first-degree relatives were assessed; recruitment was extended to include additional relatives in families that contained 2 or more first degree relatives with AUDs (n = 16,848). Data collection included a psychiatric interview, neurophysiological and neuropsychological protocols, and collection of blood for DNA. We currently have genome wide data on 12,145 individuals (8,038 individuals of European ancestry; 3,655 individuals of African ancestry). In 2004, COGA began the prospective study of adolescents and young adults, targeting assessment of youth aged 12-22 from COGA families where at least one parent had been interviewed. These subjects were re-assessed every two years; currently, 89% of individuals have 2+ interviews. COGA is *racially/ethnically diverse* (60.6% non-Hispanic White, 24.9% African American, 11.1% Hispanic, and 3.4% Other).

## Polygenic Score Creation

### Adjustment of GWAS effect sizes for linkage disequilibrium (LD)

We adjusted GWAS effect sizes for the non-independence of nearby SNPs in the genome (referred to as linkage disequilibrium, or LD) using PRS-CS ^60^. PRS-CS employs a Bayesian continuous shrinkage parameter to correct for LD using the 1000 Genomes European (1KG EUR) sample as a reference panel. In keeping with the recommendations of the developers, we utilized the 1KG EUR files distributed with the software and restricted to the ~1.3 million SNPs in the high-quality consensus genotype set defined by the HapMap 3 Consortium ^60,61^. We generated polygenic scores using HapMap 3 SNPs that overlapped with the 1KG EUR panel and target samples (~1 SNPs in both COGA and Add Health). We applied the default parameters which allows PRS-CS to estimate the optimum global shrinkage parameter.

### Polygenic scores

We computed polygenic scores (in individuals of European ancestries only^[[1]](#footnote-1)^) from the weighted sum of the effect-coded alleles for a given individual *i*:

$$S_{i}= \sum_{j = 1}^{M} \hat{\beta}_{j}g_{ij}$$

where $S_{i}$ is the polygenic score, $\hat{\beta}_{j}$ is the estimated additive effect of the effect-coded allele at SNP *j*, and $g_{ij}$ is the genotype at SNP *j*. The polygenic scores were standardized within each study cohort.

### Substance Use/Substance Use Disorder (SUD) Analyses

Our choice of statistical model for the substance use/SUD analyses depended on (1) the distribution of the phenotype and (2) the structure of the data in the study cohort (independent vs. clustered or genetically related observations). In Add Health, the vast majority of the study participants are unrelated. Therefore, we analyzed one randomly drawn individual from any related pair (pairwise KING coefficient ≥ 0.0442). We used ordinary least squares (OLS) for continuous or ordinal outcomes, and logistic regression for binary outcomes. With OLS, we evaluated the traditionally defined coefficient of determination (*R^2^*). In the case of logistic regression, we evaluated Nagelkerke’s pseudo-*R^2^* ^(62)^. COGA is a family-based cohort study with a variety of different pedigree structures ^63,64^. To adjust for familial clustering, we utilized linear mixed models for continuous and ordinal outcomes (LMM) or generalized linear mixed models (GLMM) with a logistic link function for binary outcomes. For the LMM/GLMM models, we evaluated a pseudo-*R^2^* designed specifically for mixed models ^65^.

In Add Health (*N* = 5,107), for each phenotype ($Y$), we performed two regressions to estimate the accuracy of the polygenic score in explaining phenotypic variation. Specifically, we analyzed regression equations of the following form:

| *Baseline model:* | $Y=X\beta+\varepsilon$ |
| --- | --- |
| *Polygenic score model:* | $Y=S\gamma+X\beta+\varepsilon$ |

where $S$ and $X$ are matrices for the polygenic score and covariates with corresponding vectors of regression coefficients to be estimated, $\gamma$ and $\beta$, respectively. The baseline model included covariates for sex, age, and genetic principal components (PCs), as well as genotyping batch when applicable.

In COGA (*N* = 7,483), we estimated the following regression equations:

| *Baseline model:* | $Y=X\beta+Z\mu+\varepsilon$ |
| --- | --- |
| *Polygenic score model:* | $Y=S\gamma+X\beta+Z\mu+\varepsilon$ |

where we also included a matrix $Z$ with a binary indicator for each family unit and vector of unobserved random effects $\mu$ (specified as a variance component of the error term) ^66^. The accuracy of the PGS was defined as the difference in *R^2^* (or pseudo-*R^2^*) between the two models, a measure that is sometimes called “incremental *R^2^*” (or Δ*R^2^*) ^67^.

### Longitudinal Growth Models

To assess the associations between polygenic scores and change in alcohol-related behaviors over time, we fit a series of linear mixed-effects models with the data from four waves of Add Health, structured on age. In the notation of the multilevel model would appear as:

| *Level 1:* | $Y_{ti}=\pi_{0i}+\pi_{1i}*\mathrm{Age}_{ti}+\varepsilon_{ti}$ |
| --- | --- |
| *Level 2 (intercept):* | $\pi_{0i}= \beta_{00}+ {\Sigma_{k=1} \beta}_{0k}*X_{ki}+ \mu_{0i}$ |
| *Level 2 (slope):* | $\pi_{1i}= \beta_{10}+ {\Sigma_{k=1} \beta}_{1k}*X_{ki}+ \mu_{1i}$ |

The model for within-individual change (Level 1) in AUI for time *t* of respondent *i* is composed of the respondents’ baseline alcohol misuse (*π_0i_*), the change in AUI as a function of age (*π_1i_*), and any residual variance (*e_ti_*). The Level 2 model, consisting of the between-individual portion (with a single, linear slope) includes the influence of *k* number of covariates (X's) on baseline values and change over time. Finally, we allow for random effects in the intercept and slope parameters (*u*_0_*_i_* and *u*_1_*_i_*) which have the variance-covariance matrix, *μ* = $\left[ \begin{matrix} \tau_{1}^{2} & \tau_{21} \\ \tau_{12} & \tau_{2}^{2} \end{matrix} \right]$,  and are assumed to ~*N*(0,  *μ*) and uncorrelated with the fixed-effects in the model. The linear mixed model is well suited to handle longitudinal data that is unbalanced and/or has unique response schedule^68,69^. The model fitting process proceeded as follows:

1. Determine the functional form that the alcohol use index (AUI) followed over of time through both visual inspection and fitting linear and higher order polynomial models.
2. Determine the appropriate structure of the random effects and residual components of the model using nested models.
3. Examine whether polygenic scores for EXT and ALCP-specific were associated with trajectories of AUI using both individual parameter estimates and improvements in overall model fit.

We included the effects of PGS and sex on both initial status and change over time because prior evidence of differences in trajectories across sex ^70,71^. We also examined the possibility of sex differences by fitting sex-stratified models. All other covariates were included for their effect on initial status only to limit the number of parameters. The age categories at the ends of the age spectrum were collapsed due to small cell sizes (age ranges from 12 and below, to 32 and above).

## Phenotype definitions

### Substance use

For substance use, we created measures of ever use for a variety of substances using each wave/interview in each sample. Respondents were classified as yes on *lifetime smoking initiation* if they responded yes to “Have you ever smoked cigarettes regularly, that is, at least 1 cigarette every day for 30 days?” at any point in Add Health, or yes to “Over your lifetime, have you smoked a total of 100 cigarettes (smoked 5 or more packs)?” at any point in COGA. The definition of *lifetime smoking initiation* and *cigarettes per day* in UKB has been described elsewhere^18^. *Lifetime alcohol use* was coded as yes if participants responded yes to “Have you had a drink of beer, wine, or liquor--not just a sip or taste of someone else's drink--more than 2 or 3 times in your life?” at any wave in Add Health or yes to either “Have you ever had a drink of alcohol?” or “So, you have never had even one full drink of alcohol?” in COGA. For cannabis use, participants were coded as a yes on *lifetime cannabis use* in Add Health or COGA if they responded yes to questions regarding ever using marijuana or hashish across any of the waves/interviews. Participants were classified as a lifetime opioid user (*lifetime opioid use*, COGA only) if they indicated opioids (among a list of many possible substances) for the question “Have you ever used any of these drugs to feel good or high, or to feel more active or alert? Or did you use any prescription drugs when they were not prescribed, or more than prescribed?” Finally, *lifetime other substance use* indicates whether participants have indicated they had ever used a variety of other illicit drugs or prescription medications outside their intended use. In Add Health this included ever using sedatives, tranquilizers, stimulants, painkillers, steroids, cocaine, crystal meth, and/or some other illicit substance. In COGA, the list of other substances included cocaine, stimulants, and/or sedatives.

### Substance use disorders

In addition to substance use, we created measures of substance use disorders and/or problematic use, as the genetic overlap between use and problems is only partial^11^. Both Add Health and COGA contained some form of clinical interview^72,73^. We constructed measures substance use disorders that correspond to the substances as described above. In Add Health, *alcohol use disorder (AUD) symptoms*, *cannabis use disorder (CUD) symptoms*, and *other substance use disorder (other SUD) symptoms* were measured from the combined criteria counts of DSM-IV dependence and abuse of each of their corresponding substances. The total range of possible criteria ranged from 0 to 11. In COGA, *alcohol use disorder symptoms*, *cannabis use disorder symptoms*, *opioid use disorder (OUD) symptoms*, and *other substance use disorder symptoms* were measured from criteria counts of DSM-5 substance use disorder symptoms. These responses again ranged from 0 to 11. The only differences between combining abuse and dependence from DSM-IV criteria and using the DSM-5 criteria (which mostly reflects the combination of abuse and dependence into a single disorder) are in a single item. DSM-IV abuse contains the criteria of “[i]n the past year, have you more than once gotten arrested, been held at a police station, or had other legal problems because of your drinking?” which was not included in DSM-5. Instead, DSM-5 added, “[i]n the past year, have you wanted to drink so badly you couldn’t think of anything else.” Finally, in both Add Health and COGA, we used the Fagerstrom test for nicotine dependence (FTND) to assess *nicotine dependence symptoms*. The FTND assesses six criteria and has values ranging from 0 to 10. Overall, these measures of substance use disorders provide good coverage of problematic substance use.

### Alcohol Use Index (AUI)

For the longitudinal growth models in Add Health, we created a composite measure of alcohol use/misuse across Wave I through Wave IV. This composite index included drinking frequency, drinking quantity, frequency of binge drinking, frequency of drinking to intoxication, and alcohol-related problems (e.g., problem with social relationships or commitments). Below are the specific questions and their responses:

1. *Drinking quantity* was assessed from the question: “Think of all the times you had a drink during the past 12 months. How many drinks did you usually have each time?”
2. *Drinking frequency* was assessed from the question: “During the past 12 months, how often did you drink beer, wine, or liquor?” Responses ranged from 0 = “Never” to 6 = “Every day or almost every day”.
3. *Binge drinking* was assessed from the question: “Over the past 12 months, on how many days did you drink 5 or more drinks in a row?” Responses ranged from 0 = “Never” to 6 = “Every day or almost every day”.
4. *Intoxication frequency* was assessed from the question: “During the past 12 months, on how many days have you been drunk or very high on alcohol?” Responses ranged from 0 = “Never” to 6 = “Every day or almost every day”.
5. *Alcohol Problems* was a composite assessed from the following questions at each wave (note: because the response options changed from Waves I-III to Wave IV, we recoded the outcomes, so each problem is coded 0=”Never”, 1=”Once”, and 2=”2+ times”):
   1. Wave I:
      1. “In the past 12 months: You got into trouble with your parents because you had been drinking”.
      2. “In the past 12 months: You’ve had problems at school or with schoolwork because you had been drinking”.
      3. “In the past 12 months: You had problems with your friends because you had been drinking”.
      4. “In the past 12 months: You had problems with someone you were dating because you had been drinking”.
   2. Wave II:
      1. “In the past 12 months: You got into trouble with your parents because you had been drinking”.
      2. “In the past 12 months: You’ve had problems at school or with schoolwork because you had been drinking.”.
      3. “In the past 12 months: You had problems with your friends because you had been drinking.”.
      4. “In the past 12 months: You had problems with someone you were dating because you had been drinking.”
   3. Wave III:
      1. “In the past 12 months: You had problems at school or work because you had been drinking”.
      2. “In the past 12 months: You had problems with your friends because you had been drinking”.
      3. “In the past 12 months: You had problems with someone you were dating because you had been drinking”.
      4. “How many times did you get into a sexual situation that you later regretted because you had been drinking?” or “How many times did you get into a physical fight because you had been drinking?” or “How many times did you show up to work/school drunk?”
   4. Wave IV:
      1. “How often has your drinking interfered with your responsibilities at work or school?”
      2. “How often have you been under the influence of alcohol when you could have gotten yourself or others hurt, or put yourself or others at risk, including unprotected sex?”
      3. “How often have you had legal problems because of your drinking, like being arrested for disturbing the peace or driving under the influence of alcohol, or anything else?”
      4. How often have you had problems with your family, friends, or people at work or school because of your drinking?”

All responses were coded so higher levels indicated greater levels of that alcohol-related behavior). Each of these items was then scaled to a value of 0 to 10 and averaged at each wave to create a composite measure ranging from normative use to problematic use. This method has been used in prior longitudinal models of alcohol use/misuse ^74^.

## Results of SUD analyses in Add Health and COGA

### Ever Substance Use

Results for the PRS models are presented in **Supplementary Tables 3 & 4**. PRSs for ALCP-total were associated with cannabis use (OR_Add Health_ = 1.15, *P* = 7.14x10^-6^; OR_COGA_ = 1.20, *P* = 8.56x10^-9^), nicotine use (OR_Add Health_ = 1.09, *P* = 1.89x10^-3^; OR_COGA_ = 1.23, *P* = 2.35x10^-11^), other illicit substance use (OR_Add Health_ = 1.20, *P* = 2.24x10^-8^; OR_COGA_ = 1.27, *P* = 7.71x10^-16^), and opioid use (OR_COGA_ = 1.24, *P* = 4.09x10^-10^) in each of the samples in which they were measured, even after correcting for a false discovery rate of 5%. The only substance that the U-ALPCS PRS were not associated with was *lifetime alcohol use*, which is likely reflection of the ubiquitous nature of this phenotype (95–96% of the Add Health and COGA participants report lifetime alcohol initiation). When we compare this to results for ALCP-specific, the PRSs for ALCP-specific were only associated with cannabis use (OR_COGA_ = 1.08, *P* = 1.19x10^-2^), other illicit substance use (OR_Add Health_ = 1.12, *P* = 6.61x10^-4^; OR_COGA_ = 1.15, *P* = 1.83x10^-6^), and opioid use (OR_COGA_ = 1.12, *P* = 1.07x10^-3^) after correcting for multiple testing. In each case where ALCP-specific remained significant, the effect sizes were markedly reduced. Finally, the EXT PRS were associated with every substance use phenotype in each of the samples, even after correcting for multiple testing (ORs = 1.32 – 1.70).

### Substance Use Disorder Criterion Count

When we consider substance use disorder (SUD) criterion counts, we identified positive associations between the O-ALCP PRS and alcohol use disorder criteria (βAdd Health = 0.17, P = 3.53x10-7; βCOGA = 0.49, P = 4.62x10-29), cannabis use disorder criteria (βAdd Health = 0.11, P = 8.88x10-4; βCOGA = 0.16, P = 3.47x10-4), nicotine dependence symptoms (βCOGA = 0.20, P = 2.78x10-5), and other substance use disorder criteria (βCOGA = 0.20, P = 8.48x10-3) after correcting for multiple testing. The PRSs for ALCP-specific were associated with alcohol use disorder criteria (βAdd Health = 0.13, P = 1.53x10-4; βCOGA = 0.34, P = 5.97x10-15), cannabis use disorder criteria (βAdd Health = 0.07, P = 3.17x10-2), and nicotine dependence symptoms (βCOGA = 0.10, P = 3.62x10-2). Again, these effect sizes are attenuated between O-ALCP and ALCP-specific. If we use the more stringent Bonferroni threshold (.05/60, p < 0.000833333), only the association with AUD criteria remains. The PRS for EXT were significantly associated with each of the SUD criterion counts, even after correcting for multiple testing (β’s = 0.11 – 0.59).

Finally, when we compare the variance explained by ALCP-total to that from adding the externalizing and the alcohol specific PGS (EXT + ALCP-specific) jointly to the baseline model, we see an improvement in predictive power in both Add Health (Δ$R_{EXT+ALCP-S}^{2}$ = 0.98%; β_EXT_ = 0.20, SE_EXT_ = 0.03, P_EXT_ = 3.83x10-9; β_ALCP-specific_ = 0.14, SE_ALCP-specific_ = 0.03, P_ALCP-specific_ = 5.63x10-5) and COGA (Δ$R_{EXT+ALCP-S}^{2}$ = 3.13%; β_EXT_ = 0.58, SE_EXT_ = 0.05, P_EXT_ = 9.66x10-39; β_ALCP-specific_ = 0.35, SE_ALCP-specific_ = 0.04, P_ALCP-specific_ = 1.14x10-15), which was comparable to adding the ALCP-total and EXT PGS (Add Health Δ$R_{EXT+ALCP-O}^{2}$ = 0.93%; COGA Δ$R_{EXT+ALCP-O}^{2}$ = 3.21%). Because the PGS for EXT and ALCP-specific are uncorrelated by design, the overall predictive power of these two scores is approximately the sum of the two. The overall predictive power of ALCP-total and EXT PGS is less than their sum as these two scores are correlated (r ~ 0.24 in both Add Health and COGA).

## Results of Longitudinal Growth Models in Add Health

### Initial model building

We followed the steps for model building outlined in section 5.2.4, above. Results for the initial models are presented in **Supplementary Table 5**. We first ran an unconditional model (intercept only) to estimate the intraclass correlation coefficient (ICC), which provides an estimate of the degree of variance explained by between (vs within) cluster variance. For our alcohol use index (AUI), we estimated an ICC = .263, suggesting there is substantial within-person variance in AUI over time. In order to determine the functional form of change over time, we first plotted the mean levels of AUI by age and sex in the full AUI and each of its component indicators (see below).

Supplementary Figure 5 Mean Levels of Alcohol Phenotypes Over Time in Add Health

Mean levels of drinking frequency (Freq), drinking quantity (Drinks), binge drinking (HED), drinking to intoxication (Intox), alcohol-related problems (Probs), and alcohol use index (AUI) from ages 12 to 32 in Add Health (N = 5,107).

For each of the AUI indicators and the AUI composite score, a quadratic change over time seemed to best describe the data. Including both linear (Δχ^2^ = 431.62, Δdf = 1, *P* = *7*.22x10^-96^) and quadratic (Δχ^2^ = 397.32, Δdf = 1, *P* = *2*.11x10^-88^) terms for age in the fixed effects portion of the model resulted in significantly better fit. We carried this model forward to estimate the structure of the random effects. Next, we evaluated several configurations of the random effects structure including random intercepts and slopes for the linear change in age (with a correlation between these two) and a random slope for both the linear and quadratic slopes of age (age and age^2^). We settled on the latter due to the better fit indices (AIC = 55158.03 vs 55356.60) and the fact that the estimates for baseline values in AUI were very close to zero. We selected this quadratic model with random effects for age and age^2^ as well as the covariance between the two) to test whether the EXT and ALCP-specific PRSs were associated with baseline levels or change over time in AUI.

### Longitudinal Models of Polygenic Risk and Alcohol Misuse

Using the above model as our baseline model, we tested the association between polygenic scores for EXT and ALCP-specific with baseline levels and change over time in AUI. For each model, we included sex and the first ten ancestral principal components (PCs) as covariates. Beginning with EXT, we tested the associations in a stepwise approach, first adding the EXT PRS in the first model, and then interaction between the EXT PRS and age, followed by the interaction between the EXT PRS and age^2^. Including the EXT PRS in the model for baseline AUI significantly improved the fit of the model (Δχ^2^ = 69.20, Δdf = 1, *P* = 8.89x10^-17^), while including the EXT PRS in the model for linear change (Δχ^2^ = 0.49, Δdf = 1, *P* = 4.82x10^-1^) or quadratic change (Δχ^2^ = 0.02, Δdf = 1, *P* = 8.87x10^-1^) did not improve the fit of the model. We therefore only carried forward the effect of EXT PRS on baseline AUI in the models testing the ALCP-specific PRS and followed the same stepwise approach. ALCP-specific significantly improved fit for both the baseline (Δχ^2^ = 13.48, Δdf = 1, *P* = 2.41x10^-4^) and linear change (Δχ^2^ = 19.59, Δdf = 1, *P* = 9.59x10^-6^) models. The overall model that best describes the relationship between the EXT and ALCP-specific PRSs and change in AUI over time is one in which those with higher levels of EXT have greater levels of AUI at baseline, and those with higher ALCP-specific have both a greater AUI at baseline and a steeper increase in AUI over time.

Supplementary Figure 6: Predicted Trajectories of AUI Across Age, Sex, and Polygenic Risk

Predicted values for alcohol use index (AUI) from ages 12 to 32 using linear mixed models in Add Health (N=5,107). Values for EXT and ALCP-specific polygenic scores set to ±1.5 SD. The shaded areas represent 95% confidence intervals. Confidence intervals estimated using percentile method bootstrapping over 1000 bootstrap samples. All other covariates set to mean values.

The final tests we performed were to assess potential sex differences in trajectories of AUI, especially given the differences in mean levels seen in plotting the observed values. We tested two potential models for sex differences. In the first model, we allowed the effect of change over time to vary by sex (e.g., inclusion of sex-by-age and sex-by-age^2^ interactions). In the second model we also allowed the effect of each PRS to vary by sex (e.g., inclusion of sex-by-PRS, sex-by-PRS-by-age, and sex-by-PRS-by-age^2^ interactions). Full parameter estimates for these models are available in **Supplementary Table 6**. Including sex differences in change over time resulted in a substantial increase in model fit (Δχ^2^ = 244.58, Δdf = 2, *P* = 7.77x10^-54^). Including the interactions with PRS also significantly improved model fit (Δχ^2^ = 18.28, Δdf = 3, *P* = 3.85x10^-4^). However, given that only one of the interactions was significant (sex*age*ALCP-specific PRS), and the relatively marginal significant of this interaction (*β* = 0.070, *P* = 4.58x10^-2^), we did not select the model with full sex differences and instead selected the first model with sex differences in change over time, only. The figure below provides predicted trajectories across sex, PRS (± 1.5 SD of both EXT and ALCP-specific PRS), and age.

Lastly, we compared the results from the main longitudinal models to the same models using the ALCP-total PGS only. In the table below, we see that that the effect of ALCP-total on the slope of linear change is similar to the results for the association between the ALCP-specific and linear change in AUI over time. The ALCP-total PGS is more similar to the ALCP-specific PGS than the EXT PGS in terms for the association with baseline values in the alcohol use index. Finally, there was no significant interaction between the ALCP-total PGS and the function for quadratic change.

| **Comparison of EXT+ALCP-specific vs ALCP-total Longitudinal Results** | | | | | | | | | |
| --- | --- | --- | --- | --- | --- | --- | --- | --- | --- |
| **Final model for EXT + ALCP-specific** | | | | | | | | | |
|  | Baseline | | | Linear change | | | Quadratic change | | |
| Covariate | Beta | SE | P | Beta | SE | P | Beta | SE | P |
| Int | **0.457** | **0.044** | **7.70E-25** | **1.200** | **0.111** | **2.31E-27** | **-0.513** | **0.053** | **7.13E-22** |
| Male | **-0.370** | **0.066** | **2.41E-08** | **1.657** | **0.163** | **2.94E-24** | **-0.572** | **0.078** | **2.04E-13** |
| EXT PGS | **0.113** | **0.013** | **1.66E-17** | - | - | - | - | - | - |
| ALCP-specific PGS | 0.002 | 0.019 | 9.05E-01 | **0.064** | **0.018** | **2.48E-04** | - | - | - |
|  |  |  |  |  |  |  |  |  |  |
|  |  |  |  |  |  |  |  |  |  |
| **Final Model for ALCP-total** | | | | | | | | | |
|  | Baseline | | | Linear change | | | Quadratic change | | |
| *Covariate* | *Beta* | *SE* | *P* | *Beta* | *SE* | *P* | *Beta* | *SE* | *P* |
| Int | **0.468** | **0.044** | **6.14E-26** | **1.186** | **0.111** | **1.09E-26** | **-0.508** | **0.053** | **2.13E-21** |
| Male | **-0.377** | **0.066** | **1.32E-08** | **1.660** | **0.163** | **2.78E-24** | **-0.573** | **0.078** | **1.95E-13** |
| ALCP-total PGS | 0.006 | 0.019 | 7.66E-01 | **0.078** | **0.018** | **9.47E-06** | - | - | - |
|  |  |  |  |  |  |  |  |  |  |
| Models also included ancestral principal components as covariates (for baseline only) | | | | | | | | | |
| Bolded estimates = *p* < .05, Int = baseline levels of AUI, linear change, and quadratic change (intercept, age, and age-squared) | | | | | | | | | |

# References

1 Levey DF, Stein MB, Wendt FR, Pathak GA, Zhou H, Aslan M *et al.* Bi-ancestral depression GWAS in the Million Veteran Program and meta-analysis in >1.2 million individuals highlight new therapeutic directions. *Nat Neurosci* 2021. doi:10.1038/s41593-021-00860-2.

2 Howard DM, Adams MJ, Clarke TK, Hafferty JD, Gibson J, Shirali M *et al.* Genome-wide meta-analysis of depression identifies 102 independent variants and highlights the importance of the prefrontal brain regions. *Nat Neurosci* 2019; **22**: 343–352.

3 Mullins N, Forstner AJ, O’Connell KS, Coombes B, Coleman JRI, Qiao Z *et al.* Genome-wide association study of more than 40,000 bipolar disorder cases provides new insights into the underlying biology. *Nat Genet* 2021. doi:10.1038/s41588-021-00857-4.

4 Levey DF, Gelernter J, Polimanti R, Zhou H, Cheng Z, Aslan M *et al.* Reproducible Genetic Risk Loci for Anxiety: Results From ∼200,000 Participants in the Million Veteran Program. *Am J Psychiatry* 2020. doi:10.1176/appi.ajp.2019.19030256.

5 Nievergelt CM, Maihofer AX, Klengel T, Atkinson EG, Chen CY, Choi KW *et al.* International meta-analysis of PTSD genome-wide association studies identifies sex- and ancestry-specific genetic risk loci. *Nat Commun* 2019. doi:10.1038/s41467-019-12576-w.

6 Walters RK, Polimanti R, Johnson EOECEO, McClintick JN, Adams MJ, Adkins AE *et al.* Trans-ancestral GWAS of alcohol dependence reveals common genetic underpinnings with psychiatric disorders. *Nat Neurosci* 2018; **21**: 1656–1669.

7 Zhou H, Rentsch CT, Cheng Z, Kember RL, Nunez YZ, Sherva RM *et al.* Association of OPRM1 Functional Coding Variant With Opioid Use Disorder: A Genome-Wide Association Study. *JAMA Psychiatry* 2020. doi:10.1001/jamapsychiatry.2020.1206.

8 Johnson EC, Demontis D, Thorgeirsson TE, Walters RK, Polimanti R, Hatoum AS *et al.* A large-scale genome-wide association study meta-analysis of cannabis use disorder. *Lancet Psychiatry* 2020. doi:10.1016/S2215-0366(20)30339-4.

9 Trubetskoy V, Pardiñas AF, Qi T, Panagiotaropoulou G, Awasthi S, Bigdeli TB *et al.* Mapping genomic loci implicates genes and synaptic biology in schizophrenia. *Nature 2022* 2022; : 1–13.

10 Anttila V, Bulik-Sullivan B, Finucane HK, Walters RK, Bras J, Duncan L *et al.* Analysis of shared heritability in common disorders of the brain. *Science (1979)* 2018; **360**: eaap8757.

11 Sanchez-Roige S, Palmer AA, Clarke TK. Recent Efforts to Dissect the Genetic Basis of Alcohol Use and Abuse. Biol Psychiatry. 2020. doi:10.1016/j.biopsych.2019.09.011.

12 Kendler KS, Myers J. The boundaries of the internalizing and externalizing genetic spectra in men and women. *Psychol Med* 2014; **44**: 647–655.

13 Kendler KS, Prescott CA, Myers J, Neale MC. The structure of genetic and environmental risk factors for common psychiatric and substance use disorders in men and women. *Arch Gen Psychiatry* 2003; **60**: 929–937.

14 Lahey BB, Rathouz PJ, van Hulle C, Urbano RC, Krueger RF, Applegate B *et al.* Testing structural models of DSM-IV symptoms of common forms of child and adolescent psychopathology. *J Abnorm Child Psychol* 2008; **36**: 187–206.

15 Lahey BB, Waldman ID, Hankin BL, Applegate B, Loft JD, Rick J. The structure of child and adolescent psychopathology: Generating new hypotheses. *J Abnorm Psychol* 2004; **113**: 358–385.

16 Krueger RF, Markon KE, Patrick CJ, Benning SD, Kramer MD. Linking antisocial behavior, substance use, and personality: an integrative quantitative model of the adult externalizing spectrum. *J Abnorm Psychol* 2007; **116**: 645.

17 Krueger RF, Hicks BM, Patrick CJ, Carlson SR, Iacono WGWG, McGue M. Etiological connections among substance dependence, antisocial behavior and personality: Modeling the externalizing spectrum. *J Abnorm Psychol* 2002; **111**: 411–424.

18 Karlsson Linnér R, Biroli P, Kong E, Meddens SFW, Wedow R, Fontana MA *et al.* Genome-wide association analyses of risk tolerance and risky behaviors in over 1 million individuals identify hundreds of loci and shared genetic influences. *Nat Genet* 2019; **51**: 245–257.

19 Young SE, Stallings MC, Corley RP, Krauter KS, Hewitt JK. Genetic and environmental influences on behavioral disinhibition. *Am J Med Genet* 2000; **96**: 684–695.

20 King SM, Iacono WG, Mcgue M. Childhood externalizing and internalizing psychopathology in the prediction of early substance use. *Addiction* 2004; **99**: 1548–1559.

21 Young SE, Friedman NP, Miyake A, Willcutt EG, Corley RP, Haberstick BC *et al.* Behavioral Disinhibition: Liability for Externalizing Spectrum Disorders and Its Genetic and Environmental Relation to Response Inhibition Across Adolescence. *J Abnormal Psychol* 2009; **118**: 117–130.

22 Kotov R, Cicero DC, Conway CC, DeYoung CG, Dombrovski A, Eaton NR *et al.* The Hierarchical Taxonomy of Psychopathology (HiTOP) in psychiatric practice and research. *Psychol Med* 2022; : 1–13.

23 Hicks BM, Krueger RF, Iacono WG, McGue M, Patrick CJ. Family transmission and heritability of externalizing disorders: A Twin-Family Study. *Arch Gen Psychiatry* 2004; **61**: 922–928.

24 Kendler KS, Chen X, Dick DM, Maes HH, Gillespie NA, Neale MC *et al.* Recent advances in the genetic epidemiology and molecular genetics of substance use disorders. *Nat Neuroscience* 2012; **15**: 181–189.

25 Barr PB, Ksinan A, Su J, Johnson EC, Meyers JL, Wetherill L *et al.* Using polygenic scores for identifying individuals at increased risk of substance use disorders in clinical and population samples. *Transl Psychiatry* 2020; **10**: 196.

26 Salvatore JE, Aliev F, Bucholz K, Agrawal A, Hesselbrock V, Hesselbrock M *et al.* Polygenic Risk for Externalizing Disorders: Gene-by-Development and Gene-by-Environment Effects in Adolescents and Young Adults. *Clinical Psychological Science* 2015; **3**: 189–201.

27 Karlsson Linnér R, Mallard TT, Barr PB, Sanchez-Roige S, Madole JW, Driver MN *et al.* Multivariate analysis of 1.5 million people identifies genetic associations with traits related to self-regulation and addiction. *Nat Neurosci* 2021; : 1–10.

28 SAUNDERS JB, AASLAND OG, BABOR TF, DE LA FUENTE JR, GRANT M. Development of the Alcohol Use Disorders Identification Test (AUDIT): WHO Collaborative Project on Early Detection of Persons with Harmful Alcohol Consumption‐II. *Addiction* 1993; **88**: 791–804.

29 Sanchez-Roige S, Palmer AA, Fontanillas P, Elson SL, Adams MJ, Howard DM *et al.* Genome-wide association study meta-analysis of the alcohol use disorders identification test (AUDIT) in two population-based cohorts. *American Journal of Psychiatry* 2019; **176**: 107–118.

30 Loh P-R, Tucker G, Bulik-Sullivan BK, Vilhjálmsson BJ, Finucane HK, Salem RM *et al.* Efficient Bayesian mixed-model analysis increases association power in large cohorts. *Nat Genet* 2015; **47**: 284–290.

31 Loh P-R, Kichaev G, Gazal S, Schoech AP, Price AL. Mixed-model association for biobank-scale datasets. *Nat Genet* 2018; **50**: 906–908.

32 Bycroft C, Freeman C, Petkova D, Band G, Elliott LT, Sharp K *et al.* The UK Biobank resource with deep phenotyping and genomic data. *Nature* 2018; **562**: 203–209.

33 Willer CJ, Li Y, Abecasis GR. METAL: fast and efficient meta-analysis of genomewide association scans. *Bioinformatics* 2010; **26**: 2190–2191.

34 Grotzinger AD, Rhemtulla M, de Vlaming R, Ritchie SJ, Mallard TT, Hill WD *et al.* Genomic structural equation modelling provides insights into the multivariate genetic architecture of complex traits. *Nat Hum Behav* 2019; **3**: 513–525.

35 Benjamini Y, Hochberg Y. Controlling the False Discovery Rate: A Practical and Powerful Approach to Multiple Testing. *Journal of the Royal Statistical Society Series B (Methodological)* 1995; **57**: 289–300.

36 Watanabe K, Taskesen E, van Bochoven A, Posthuma D. Functional mapping and annotation of genetic associations with FUMA. *Nat Commun* 2017; **8**: 1–11.

37 de Leeuw CA, Mooij JM, Heskes T, Posthuma D. MAGMA: Generalized Gene-Set Analysis of GWAS Data. *PLoS Comput Biol* 2015; **11**: e1004219.

38 Liberzon A, Subramanian A, Pinchback R, Thorvaldsdóttir H, Tamayo P, Mesirov JP. Molecular signatures database (MSigDB) 3.0. *Bioinformatics* 2011; **27**: 1739–1740.

39 Sey NYA, Hu B, Mah W, Fauni H, McAfee JC, Rajarajan P *et al.* A computational tool (H-MAGMA) for improved prediction of brain-disorder risk genes by incorporating brain chromatin interaction profiles. 2020.

40 Wang D, Liu S, Warrell J, Won H, Shi X, Navarro FCP *et al.* Comprehensive functional genomic resource and integrative model for the human brain. *Science (1979)* 2018; **362**. doi:10.1126/science.aat8464.

41 Won H, De La Torre-Ubieta L, Stein JL, Parikshak NN, Huang J, Opland CK *et al.* Chromosome conformation elucidates regulatory relationships in developing human brain. *Nature* 2016; **538**: 523–527.

42 Rajarajan P, Borrman T, Liao W, Schrode N, Flaherty E, Casiño C *et al.* Neuron-specific signatures in the chromosomal connectome associated with schizophrenia risk. *Science (1979)* 2018; **362**. doi:10.1126/science.aat4311.

43 Barbeira AN, Dickinson SP, Bonazzola R, Zheng J, Wheeler HE, Torres JM *et al.* Exploring the phenotypic consequences of tissue specific gene expression variation inferred from GWAS summary statistics. *Nat Commun* 2018; **9**: 1825.

44 Lonsdale J, Thomas J, Salvatore M, Phillips R, Lo E, Shad S *et al.* The Genotype-Tissue Expression (GTEx) project. *Nat Genet* 2013; **45**: 580–585.

45 Turcot V, Lu Y, Highland HM, Schurmann C, Justice AE, Fine RS *et al.* Protein-altering variants associated with body mass index implicate pathways that control energy intake and expenditure in obesity. *Nat Genet* 2018; **50**: 26–35.

46 Karlsson T, Rask-Andersen M, Pan G, Höglund J, Wadelius C, Ek WE *et al.* Contribution of genetics to visceral adiposity and its relation to cardiovascular and metabolic disease. *Nat Med* 2019; **25**: 1390–1395.

47 Pulit SL, Stoneman C, Morris AP, Wood AR, Glastonbury CA, Tyrrell J *et al.* Meta-Analysis of genome-wide association studies for body fat distribution in 694 649 individuals of European ancestry. *Hum Mol Genet* 2019; **28**: 166–174.

48 Luciano M, Hagenaars SP, Davies G, Hill WD, Clarke TK, Shirali M *et al.* Association analysis in over 329,000 individuals identifies 116 independent variants influencing neuroticism. *Nat Genet* 2018; **50**: 6–11.

49 Nagel M, Watanabe K, Stringer S, Posthuma D, Van Der Sluis S. Item-level analyses reveal genetic heterogeneity in neuroticism. *Nat Commun* 2018; **9**: 905.

50 Zhou H, Sealock JM, Sanchez-Roige S, Clarke TK, Levey DF, Cheng Z *et al.* Genome-wide meta-analysis of problematic alcohol use in 435,563 individuals yields insights into biology and relationships with other traits. *Nat Neurosci* 2020. doi:10.1038/s41593-020-0643-5.

51 Cox AJ, Hugenschmidt CE, Raffield LM, Langefeld CD, Freedman BI, Williamson JD *et al.* Heritability and genetic association analysis of cognition in theDiabetes Heart Study. *Neurobiol Aging* 2014; **35**: 1958.e3-1958.e12.

52 Savage JE, ..., Posthuma D, Jansen PR, Stringer S, Watanabe K *et al.* Genome-wide association meta-analysis in 269,867 individuals identifies new genetic and functional links to intelligence. *Nat Genet* 2018; **50**: 912–919.

53 Jansen PR, Watanabe K, Stringer S, Skene N, Bryois J, Hammerschlag AR *et al.* Genome-wide analysis of insomnia in 1,331,010 individuals identifies new risk loci and functional pathways. *Nat Genet* 2019; **51**: 394–403.

54 Tachmazidou I, Süveges D, Min JL, Ritchie GRS, Steinberg J, Walter K *et al.* Whole-Genome Sequencing Coupled to Imputation Discovers Genetic Signals for Anthropometric Traits. *Am J Hum Genet* 2017; **100**: 865–884.

55 Day FR, Ong KK, Perry JRB. Elucidating the genetic basis of social interaction and isolation. *Nat Commun* 2018; **9**: 1–6.

56 van der Meer D, Frei O, Kaufmann T, Shadrin AA, Devor A, Smeland OB *et al.* Understanding the genetic determinants of the brain with MOSTest. *Nat Commun* 2020; **11**: 1–9.

57 Lindström S, Wang L, Smith EN, Gordon W, Van Hylckama Vlieg A, De Andrade M *et al.* Genomic and transcriptomic association studies identify 16 novel susceptibility loci for venous thromboembolism. *Blood* 2019; **134**: 1645–1657.

58 Liu C, Kraja AT, Smith JA, Brody JA, Franceschini N, Bis JC *et al.* Meta-analysis identifies common and rare variants influencing blood pressure and overlapping with metabolic trait loci. *Nat Genet* 2016; **48**: 1162–1170.

59 Braudt DB, Mullan Harris K. Polygenic Scores (PGSs) in the National Longitudinal Study of Adolescent to Adult Health (Add Health) – Release 1. 2018.

60 Ge T, Chen C-Y, Ni Y, Feng Y-CA, Smoller JW. Polygenic prediction via Bayesian regression and continuous shrinkage priors. *Nat Commun* 2019; **10**: 1776.

61 Altshuler DM, Gibbs RA, Peltonen L, Schaffner SF, Yu F, Dermitzakis E *et al.* Integrating common and rare genetic variation in diverse human populations. *Nature* 2010; **467**: 52–58.

62 Nagelkerke NJD. A note on a general definition of the coefficient of determination. *Biometrika* 1991; **78**: 691–692.

63 Edenberg HJ. The collaborative study on the genetics of alcoholism: An update. *Alcohol Research and Health* 2002; **26**: 214–218.

64 Bucholz KK, McCutcheon V V., Agrawal A, Dick DM, Hesselbrock VM, Kramer JR *et al.* Comparison of Parent, Peer, Psychiatric, and Cannabis Use Influences Across Stages of Offspring Alcohol Involvement: Evidence from the COGA Prospective Study. *Alcohol Clin Exp Res* 2017; **41**: 359–368.

65 Nakagawa S, Schielzeth H, O’Hara RB. A general and simple method for obtaining R-squared from generalized linear mixed-effects models. *Methods Ecol Evol* 2013; **4**: 133–142.

66 Wooldridge JM. *Econometric Analysis of Cross Section and Panel Data*. The MIT Press, 2010 doi:10.2307/j.ctt5hhcfr.

67 Lee JJ, Wedow R, Okbay A, Kong E, Maghzian O, Zacher M *et al.* Gene discovery and polygenic prediction from a genome-wide association study of educational attainment in 1.1 million individuals. *Nat Genet* 2018; **50**: 1112–1121.

68 Singer JD, Willett JB. *Applied longitudinal data analysis: Modeling change and event occurrence*. Oxford university press, 2003.

69 Fitzmaurice GM, Laird NM, Ware JH. *Applied longitudinal analysis*. Wiley, 2012.

70 Chen P, Jacobson KC. Developmental trajectories of substance use from early adolescence to young adulthood: gender and racial/ethnic differences. *Journal of Adolescent Health* 2012; **50**: 154–163.

71 Barr PB. Neighborhood conditions and trajectories of alcohol use and misuse across the early life course. *Health Place* 2018; **51**: 36–44.

72 Cottler LB, Robins LN, Helzer JE. The Reliability of the CIDI-SAM: a comprehensive substance abuse interview. *Br J Addict* 1989; **84**: 801–814.

73 Bucholz KK, Cadoret R, Cloninger CR, Dinwiddie SH, Hesselbrock VM, Nurnberger JI *et al.* A new, semi-structured psychiatric interview for use in genetic linkage studies: a report on the reliability of the SSAGA. *J Stud Alcohol* 1994; **55**: 149–158.

74 Vachon DD, Krueger RF, Irons DE, Iacono WG, McGue M. Are Alcohol Trajectories a Useful Way of Identifying At-Risk Youth? A Multiwave Longitudinal-Epidemiologic Study. *J Am Acad Child Adolesc Psychiatry* 2017; **56**: 498–505.

1. Ancestry assignment was estimated from genetic data. See Braudt and Harris (2018) for full description of Add Health ancestry assignment. In COGA, ancestry was empirically assigned using the 1000 Genomes (phase 3) reference panel (YRI, CEU, JPT and CHB populations) as reference points. [↑](#footnote-ref-1)
